# Dietary restriction reprograms CD8^+^ T cell fate to enhance anti-tumour immunity and immunotherapy responses

**DOI:** 10.1038/s42255-025-01415-6

**Published:** 2025-12-09

**Authors:** Brandon M. Oswald, Lisa M. DeCamp, Joseph Longo, Michael S. Dahabieh, Nicholas Bunda, Benjamin K. Johnson, McLane J. Watson, Shixin Ma, Samuel E. J. Preston, Ryan D. Sheldon, Michael P. Vincent, Abigail E. Ellis, Molly T. Soper-Hopper, Christine Isaguirre, Dahlya Kamarudin, Hui Shen, Kelsey S. Williams, Peter A. Crawford, Susan Kaech, H. Josh Jang, Evan C. Lien, Connie M. Krawczyk, Russell G. Jones

**Affiliations:** 1 Department of Metabolism and Nutritional Programming, Van Andel Institute, Grand Rapids, MI USA; 2 Department of Epigenetics, Van Andel Institute, Grand Rapids, MI USA; 3 Salk Institute, La Jolla, CA USA; 4 Mass Spectrometry Core, Van Andel Institute, Grand Rapids, MI USA; 5 Metabolism and Nutrition (MeNu) Program, Van Andel Institute, Grand Rapids, MI USA; 6 Department of Medicine, Division of Molecular Medicine, University of Minnesota, Minneapolis, MN USA; 7 Department of Biochemistry, Molecular Biology, and Biophysics, University of Minnesota, Minneapolis, MN USA

**Keywords:** Immunosurveillance, Cancer metabolism, Immunotherapy, Metabolomics, Metabolism

## Abstract

Reducing calorie intake through dietary restriction (DR) slows tumour growth in mammals, yet the underlying mechanisms are poorly defined. Here, we show that DR enhances anti-tumour immunity by optimizing CD8^+^ T cell function within the tumour microenvironment (TME). Using syngeneic xenograft tumour models, we found that DR induces a profound reprogramming of CD8^+^ T cell fate in the TME, favouring the expansion of effector T cell subsets with enhanced metabolic capacity and cytotoxic potential, while limiting the accumulation of terminally exhausted T cells. This metabolic reprogramming is driven by enhanced ketone body oxidation, particularly β-hydroxybutyrate (βOHB), which is elevated in both the circulation and tumour tissues of DR-fed mice. βOHB fuels T cell oxidative metabolism under DR, increasing mitochondrial membrane potential and tricarboxylic acid cycle-dependent pathways critical for T cell effector function, including acetyl-CoA production. By contrast, T cells deficient for ketone body oxidation exhibit reduced mitochondrial function, increased exhaustion and fail to control tumour growth under DR conditions. Importantly, DR synergizes with anti-PD1 immunotherapy, further augmenting anti-tumour T cell responses and limiting tumour progression. Our findings reveal that T cell metabolic reprogramming is central to the anti-tumour effects of DR, highlighting nutritional control of CD8^+^ T cell fate as a key driver of anti-tumour immunity.

## Abstract (web summary)

Dietary restriction promotes the expansion of effector T cells via ketone bodies, which enhances anti-tumour immunity and synergizes with immunotherapy in mice.

## Main

The balance between cancer cell proliferation and anti-tumour functions of the immune system influences tumour growth^1^. Cytotoxic effector CD8^+^ T cells (T_eff_ cells) provide essential protective immunity against cancer^2,3^. Immune checkpoint inhibitors (ICIs) have revolutionized the treatment landscape for various malignancies, in part by promoting the expansion of CD8^+^ T cell subsets that limit tumour progression^4^. However, given their central role in controlling tumour growth, CD8^+^ T cells are often the target of immune evasion and suppression mechanisms. Chronic exposure to tumour antigens and inflammatory conditions in the TME can promote CD8^+^ T cell dysfunction (also known as exhaustion), a terminally differentiated state characterized by reduced proliferative capacity, impaired cytokine production and increased expression of inhibitory receptors (that is, PD1, LAG3, TIM3) that limit T cell effector function^5,6^. The accumulation of terminally exhausted T cells (T_ex_ cells) in tumours is driven by the transcription factor thymocyte selection-associated high mobility group box (TOX) and is reinforced through epigenetic programming^7–10^, ultimately limiting the ability of CD8^+^ T cells to control tumour growth. Understanding mechanisms that drive CD8^+^ T cell fate decisions in the TME is critical for developing new approaches to counter T cell dysfunction and overcome ICI resistance during tumour progression.

Metabolism is a key determinant of CD8^+^ T cell function and survival that underlies effective anti-tumour immunity^11^. Activated CD8^+^ T cells rewire their metabolism to produce both energy (that is, ATP production) and the biosynthetic molecules required for cell proliferation and effector function, and they use both glycolysis and oxidative phosphorylation (OXPHOS) to fuel these processes^12–14^. T cell dysfunction is also influenced by environmental conditions in the TME that impact T cell metabolism^15,16^. Chronic antigen stimulation, hypoxic stress and PD1 signalling all promote metabolic and mitochondrial derangements in CD8^+^ T cells that precede their dysfunction^17–20^. Conversely, CD8^+^ T cell effector function is enhanced by exposure to nutrients that fuel mitochondrial oxidative metabolism and acetyl-Coenzyme A (acetyl-CoA) levels, including ketone bodies and acetate^21–23^. Nutrient levels in the TME are highly influenced by diet^24^. Therefore, dietary strategies that modulate metabolic conditions in the TME have the potential to influence anti-tumour immune responses—either in isolation or in combination with ICI treatments—by metabolic reprogramming of CD8^+^ T cell function.

DR, a feeding regimen that reduces caloric intake without malnutrition, extends lifespan in mammals and delays the onset of age-related diseases, including cancer^25–29^. Dietary interventions that limit tumour growth—such as DR, fasting-mimicking diets or low glycemic diets—are presumed to act by altering growth factor (that is, insulin, IGF-1) signalling and/or nutrient availability (that is, glucose) for cancer cells^30–33^. Yet the contribution of the immune system to the anti-tumour effects of DR, either in isolation or in combination with ICI treatment, is poorly understood. Although malnutrition has deleterious effects on the immune system^34,35^, DR has been shown to enhance certain aspects of immune function^36^. Despite lower calorie intake, DR has been shown to enhance natural killer, myeloid and memory T cell responses in vivo^37–40^. Similarly, T cells isolated from calorie-restricted patients show enhanced proliferation and effector responses in vitro^41^. Here, we show that DR-driven changes in ketone body metabolism direct CD8^+^ T cell fate and function in the TME to suppress tumour growth.

## Results

### DR enhances T cell-mediated anti-tumour immunity

**Fig. 1:**
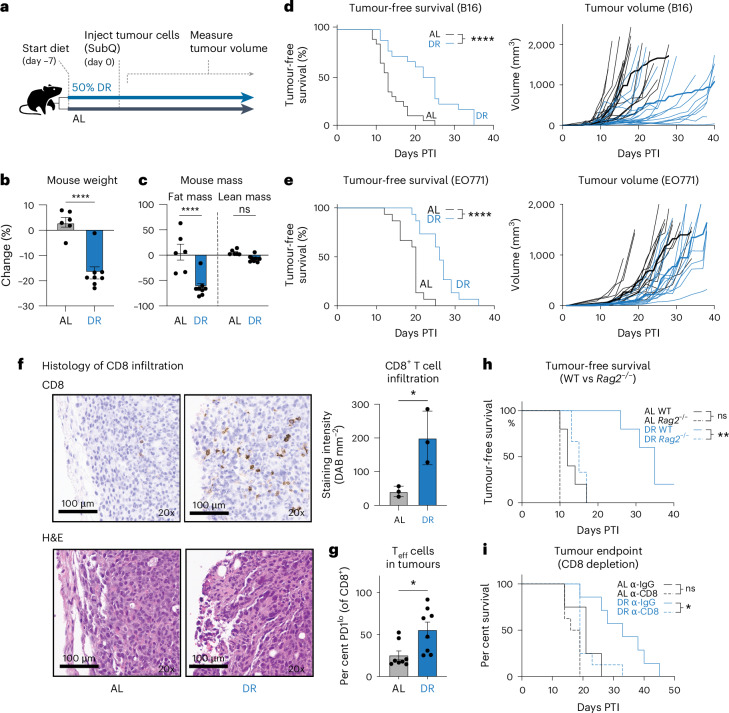
T cells mediate the anti-tumour effects of DR. **a**, Schematic representation of dietary interventions and tumour experimental design. **b**,**c**, EchoMRI analysis of mice on different dietary regimens: AL feeding or DR (50% reduction) for 2 weeks, showing the per cent change in total mouse body weight (**b**) and per cent change in fat mass and lean mass (**c**). Data are plotted as means; error bars, s.e.m. (*n* = 6 AL and *n* = 8 DR mice per group). Statistical significance was calculated with a two-tailed, unpaired Student’s *t*-test. **d**,**e**, Growth of B16 melanoma (**d**) or EO771 breast cancer cells (**e**) in C57BL/6 mice fed an AL or DR diet. Kaplan–Meier plots comparing tumour onset (tumour volume, ≥250 mm^3^) between AL and DR groups. Right panels of each graph are spaghetti plots showing growth curves of individual tumour volumes over time. The darker lines indicate the average tumour growth for each diet group over time post tumour injection (PTI). B16-OVA (melanoma), *n* =16–20 mice per group; EO771 (breast), *n* = 15 mice per group. Statistical significance was assessed by log-rank test. **f**, Histology of B16 tumours from AL-fed or DR-fed mice 14 days PTI. Left, immunohistochemical staining for CD8⁺ T cells and H&E staining of representative tumour sections. Right, quantification of positive CD8^+^ staining per tumour section (DAB per mm^2^). Data represent the means; error bars, s.e.m. (*n* = 3 mice per group). Statistical significance was calculated with a two-tailed, unpaired Student’s *t*-test. **g**, Bar graphs showing the percentage of PD1^lo^ CD8⁺ T cells (T_eff_) isolated from B16-OVA melanoma tumours, AL versus DR. Data represent the means; error bars, s.e.m. (*n* = 8 mice per group). Statistical significance was calculated with a two-tailed, unpaired Student’s *t*-test. **h**, Kaplan–Meier plot comparing tumour onset in B16 tumour-bearing WT and *Rag2*^−/−^ mice on an AL or DR diet. Statistical significance was assessed by a log-rank test (*n* = 5 mice per group). **i**, Kaplan–Meier plot comparing time-to-humane endpoint in CD8^+^ T cell-depleted mice fed an AL or DR diet. Mice were treated with control (IgG) or CD8⁺ T cell depleting (anti-CD8) antibodies before tumour cell implantation. Statistical significance was assessed by a log-rank test (*n* = 4 AL-IgG, *n* = 7 DR-IgG, *n* = 8 AL-anti-CD8, *n* = 8 DR-anti-CD8; endpoint, 1,500 mm^3^). Exact *P* values are as follows: **b**, *P* < 0.0001; **c**, fat mass, *P* < 0.0001; lean mass, *P* = 0.29; **d**, *P* < 0.0001; **e**, *P* < 0.0001; **f**, *P* = 0.0275; **g**, *P* = 0.0135; **h**, AL-WT vs DR *Rag2*^−/−^, *P* = 0.0404 (note Bonferroni correction), DR-WT vs DR *Rag2*^−/−^, *P* = 0.0042; **i**, AL (IgG) vs AL (anti-CD8), *P* = 0.0385; DR (IgG) vs DR (anti-CD8), *P* = 0.0139. *P < 0.05, **P < 0.01, ***P < 0.001, ****P < 0.0001. Source data

**Extended Data Fig. 1:**
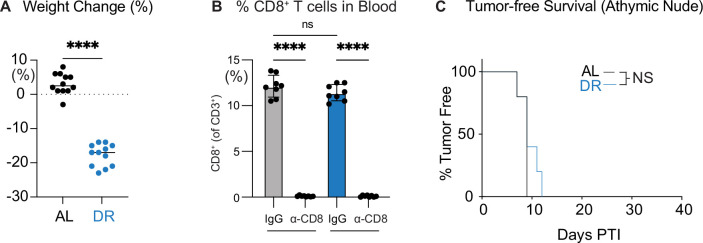
T cells mediate the anti-tumor effects of DR. **A**) Weight Change (% change from baseline) of mice after 2 weeks of feeding on AL or DR diets. Data represents the mean ± SEM (2 weeks, n = 12 mice/group). Statistical significance was calculated via two-tailed, unpaired Student’s t-test. **B**) Bar plot showing serum immunoglobulin levels in mice administered anti-IgG or anti-PD1 antibodies under AL or DR conditions. Data represent the mean ± SEM (n = 8 mice per group). Statistical significance was calculated via two-way ANOVA multiple comparisons test. **C**) Kaplan-Meier plot comparing tumor onset (tumor volume ≥250 mm^3^) in B16-OVA melanoma-bearing athymic nude mice fed ad libitum (AL) or dietary restriction (DR) diets. Statistical significance was assessed by log-rank test (n = 5 mice per group). *P < 0.05, **P < 0.01, ***P < 0.001, ****P < 0.0001. **Exact P-values are as follows:** Extended Data Fig. 1B, P = 0.0001; Extended Data Fig. 1B, AL (Ctrl) vs. AL (Anti-CD8), *P* < 0.0001; AL (Ctrl) vs. DR (Ctrl), *P* = 0.3094; AL (Ctrl) vs. DR (Anti-CD8), *P* < 0.0001; AL (Anti-CD8) vs. DR (Ctrl), *P* < 0.0001; AL (Anti-CD8) vs. DR (Anti-CD8), *P* > 0.9999; DR (Ctrl) vs. DR (Anti-CD8), *P* < 0.0001; Extended Data Fig. 1C, P = 0.2845. Source data

To assess the impact of lowering food intake on tumour growth in immunocompetent mice, we used an established model of DR in which daily food availability is reduced by 50% with no change to nutritional content (Supplementary Table 1)^38^. C57BL/6J mice were fed either ad libitum (AL) or subjected to 50% DR for 7 days before establishment of subcutaneous syngeneic tumour allografts (Fig. 1a). Consistent with previous reports^38^, this transient reduction in food intake resulted in an approximately 15–20% loss in body weight compared to AL-fed animals after 2 weeks (Fig. 1b and Extended Data Fig. 1a). Consistent with previous reports, this 50% DR produced a marked reduction in fat mass with minimal loss of lean mass after 2 weeks (Fig. 1c)^38^. Strikingly, syngeneic melanoma (B16) and breast cancer (E0771) tumours grew more slowly in mice adapted to DR, resulting in a 30–80% extension of tumour-free survival (Fig. 1d–e). These results from DR are comparable to the delay in tumour growth observed in animals fed a fasting-mimicking diet^42^.

T cells have a critical role in controlling tumour cell growth. We quantified the impact of DR on CD8⁺ T cell infiltration into syngeneic EO771 breast cancer tumours by immunohistochemistry. DR-fed mice showed a marked increase in CD8^+^ tumour-infiltrating lymphocytes (TILs) compared to AL controls (Fig. 1f). Flow cytometry analysis of TILs from B16 melanoma tumours demonstrated a similar increase in the frequency of effector CD8⁺ (PD1^lo^) T cells in mice conditioned to DR versus AL-fed mice (Fig. 1g). Strikingly, the delay in tumour growth conferred by DR was lost in *Rag2*^−/−^ mice, which lack mature T cells and B cells (Fig. 1h). Depletion of CD8^+^ T cells also abrogated the anti-tumour effects of DR (Fig. 1i and Extended Data Fig. 1b). Similarly, we observed no difference in B16 tumour growth in athymic nude mice that lack mature T cells, regardless of diet (Extended Data Fig. 1c). Together, these data indicate that the anti-tumour effects of DR require CD8⁺ T cells.

**Fig. 2:**
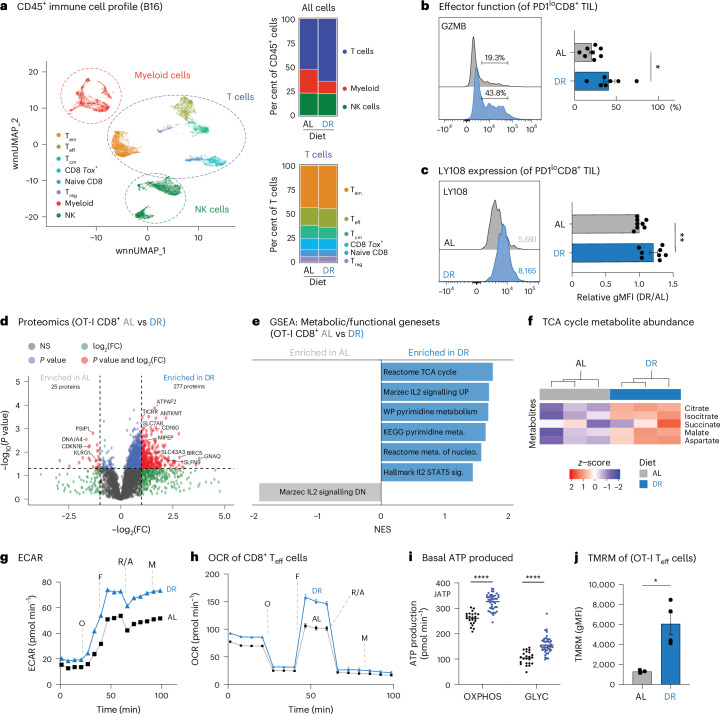
DR enhances CD8^+^ T cell effector responses and metabolic fitness in vivo. **a**, Weighted nearest-neighbour uniform manifold approximation and projection (wnnUMAP) of 45,455 CD45⁺ tumour-infiltrating cells (AL and DR combined) from B16 melanoma tumours (left; *n* = 4 mice per diet). T_em_, effector memory T cell; T_cm_, central memory T cell; T_reg_, regulatory T cell; NK, natural killer. Right, breakdown of immune cell populations from all CD45^+^ cells (top) or CD3^+^ T cells (bottom). **b**, Expression of GZMB in PD1^lo^ CD8⁺ T cells isolated from B16-OVA melanoma tumours from AL-fed or DR-fed mice. Left, representative histograms of GZMB expression; right, bar graphs quantifying the percentage of CD8^+^ cells expressing GZMB. Data represent the means; error bars, s.e.m. (*n* = 8 AL and 7 DR mice). Statistical significance was calculated with a two-tailed, unpaired Student’s *t*-test. **c**, Representative histogram of LY108 expression in PD1^lo^ CD8⁺ TILs from B16 tumours. Bar plots show relative geometric mean fluorescence intensity (gMFI) compared to AL controls. Data represent the means; error bars, s.e.m. (*n* = 8 mice per group). Statistical significance was calculated with a two-tailed, unpaired Student’s *t*-test. **d**, Mice were started on AL or DR diets 6 days before OT-I adoptive transfer. On day 0, mice were injected with *Lm*OVA in the tail vein. At 7 dpi, spleens were dissected for downstream analyses, including effector function, cell expansion and metabolic function. A volcano plot depicting the proteomics data from OT-I CD8⁺ THY1.1⁺ T cells isolated as described is shown, with log_2_(fold change (FC)) on the *x* axis and −log_10_(*P* values) on the *y* axis. Statistical significance was determined using a −log_10_(*P* value) of 1.25 and −log_2_(FC) of 1.0 as cutoff values for data visualization. **e**, GSEA focusing on metabolic and functional pathways from MSigDB (Hallmark, C2 and C5) reveals top-enriched gene sets in the DR versus AL comparison (from proteomics data, same as in **d**). Bars indicate normalized enrichment score (NES; *n* = 3 mice per group). **f**, The relative abundance (*z*-score) of TCA cycle-derived metabolites in antigen-specific CD8^+^ T cells isolated from *Lm*OVA-infected AL-fed or DR-fed mice at 7 dpi (*n* = 3 mice per group). **g**, Extracellular acidification rate (ECAR) plot of antigen-specific CD8⁺THY1.1⁺ T cells isolated from *Lm*OVA-infected mice (7 dpi) under AL or DR conditions. Data represent the means; error bars, s.d. (*n* = 24–46 technical replicates). **h**,**i**, Bioenergetic profile of AL-conditioned and DR-conditioned CD8⁺ T cells. **h**, Oxygen consumption rate (OCR) plot for antigen-specific CD8⁺ T cells isolated from *Lm*OVA-infected AL-fed or DR-fed mice (7 dpi). Data represent the means; error bars, s.d. (*n* = 24 AL and 46 DR technical replicates). Oligomycin (O), FCCP (F), rotenone and antimycin A (R/A) and monensin (M) were added to cells where indicated. **i**, Basal ATP production rates from OXPHOS and glycolysis (GLYC) for AL-conditioned or DR-conditioned CD8⁺ T cells from **h**. Data represent the means; error bars, s.d. (*n* = 24 AL and 46 DR technical replicates). Statistical significance was calculated with a two-way ANOVA multiple comparisons test. **j**, Mitochondrial membrane potential of AL-conditioned versus DR-conditioned CD8⁺ T cells. TMRM staining of CD8^+^ OT-I T cells isolated from the spleen of *Lm*OVA-infected AL-fed or DR-fed mice (7 dpi). Data represent the means; error bars, s.e.m. (*n* = 4 mice per group). Statistical significance was calculated with a two-tailed, unpaired Student’s *t*-test. *P < 0.05; **P < 0.01; ***P < 0.001; ****P < 0.0001. Exact *P* values are as follows: **b**, *P* = 0.016; **c**, *P* = 0.0042; **i**, *P* < 0.0001; **j**, *P* = 0.0143. Source data

**Extended Data Fig. 2:**
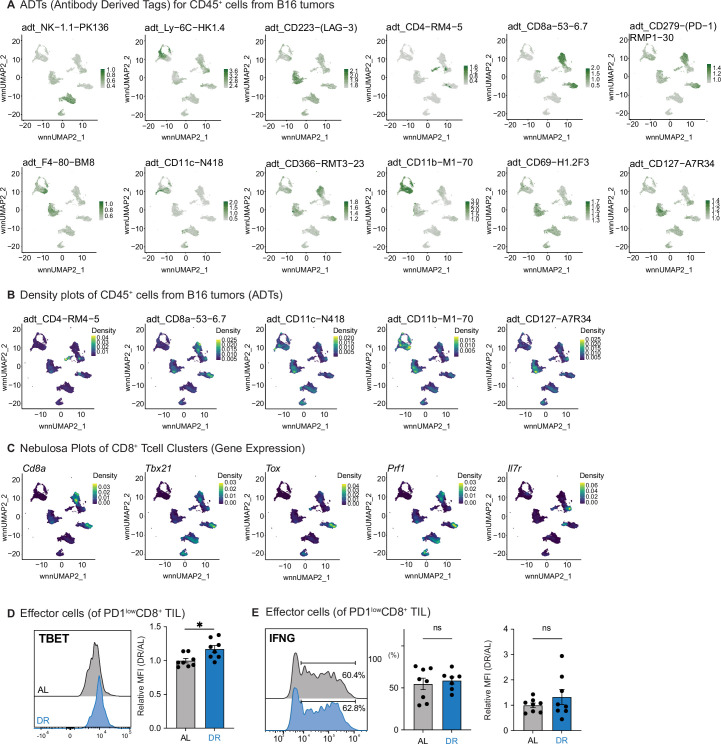
CD8^+^ T cells mediate the anti-tumor effects of DR. **A**) Weighted nearest neighbor Uniform Manifold Approximation and Projection (wnnUMAP) of 45,455 CD45⁺ tumor-infiltrating cells from B16 melanoma tumors of mice on AL or DR diets (wnnUMAP combined; n = 4 mice per diet). Each plot represents the expression of key antibody-derived tags (ADTs) for markers including NK1.1, Ly6C, LAG3 (CD223), CD4, CD8, PD1, F4/80, CD11c, TIM3 (CD366), CD11b, CD69, and CD127. **B**) Density plots generated using the Nebulosa package, showing the expression density of ADTs (CD4, CD8, CD11c, CD11b, CD127) among activated CD8⁺ T cells from B16 tumors (combined AL and DR conditions). **C**) Density plots of gene expression for *Cd8a*, *Tbx21*, *Tox*, *Prf1*, and *Il7r* among activated CD8⁺ T cells from B16 tumors (joint plot of AL and DR conditions). **D**) Representative histogram of TBET expression in PD1^Low^TOX^Low^ CD8⁺ TILs from B16 tumors. Bar plots show relative geometric mean fluorescence intensity (gMFI) compared to AL controls. Data represent the mean ± SEM (n = 8 mice per group). **E**) Representative histogram of IFN-γ expression in PD1^Low^TOX^Low^ CD8⁺ TILs from B16 tumors. Bar plots show relative gMFI compared to AL controls. Data represent the mean ± SEM (n = 8 mice per group). Statistical significance was calculated via two-tailed, unpaired Student’s t-test. *P < 0.05, **P < 0.01, ***P < 0.001, ****P < 0.0001. Source data

To determine how DR impacts immune cells in the TME, we performed cellular indexing of transcriptomes and epitopes coupled to next-generation sequencing (CITE-seq) on live CD45^+^ cells sorted from B16 tumours from AL-fed or DR-fed mice. We identified several cell type clusters that were categorized into three main groups: myeloid cells, T cells and natural killer cells (Fig. 2a, Extended Data Fig. 2a–c and Supplementary Table 2). Although recent work suggests that DR can boost natural killer cell-mediated anti-tumour immunity^39^, tumour-infiltrating natural killer cell populations were similar between dietary conditions (Fig. 2a). Notably, the proportion of tumour-infiltrating CD45^+^ T cells was increased under DR, marked by an increase in the proportion of *Gzmb*-expressing CD8^+^ T_eff_ cells (Fig. 2a–b).

To determine the impact of DR on CD8^+^ T_eff_ cell function(s) in the TME, we isolated TILs from excised B16 melanoma tumours from mice fed different diets. The majority of CD8^+^ TILs under DR conditions expressed low levels of the inhibitory receptor PD1 (Fig. 1g). Moreover, PD1^lo^ CD8^+^ TILs from B16 tumours from DR-fed mice displayed elevated TBET (Extended Data Fig. 2d) and granzyme B (GZMB) expression (Fig. 2b), consistent with heightened effector function. Although interferon-γ (IFNγ) levels in PD1^lo^ CD8^+^ TILs were similar between AL and DR conditions (Extended Data Fig. 2e), LY108/SLAMF6 expression was significantly higher in T_eff_ cells under DR, suggesting a more stem-like phenotype (Fig. 2c). Collectively, these findings indicate that DR both expands the CD8^+^ T_eff_ cell population within tumours and enhances features of cytotoxicity.

### DR enhances CD8^+^ T_eff_ cell responses and metabolic fitness in vivo

**Extended Data Fig. 3:**
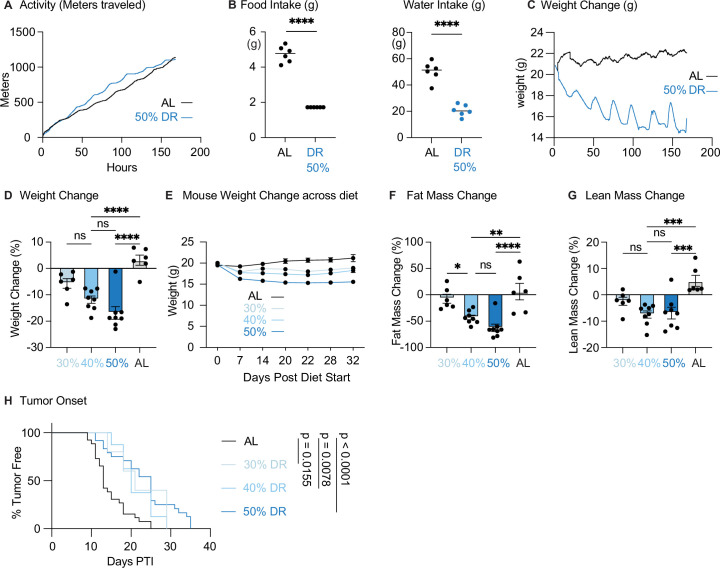
Dietary restriction reduces water intake and body weight without affecting mouse activity levels. **A**) Total distance traveled (meters) by mice on ad libitum (AL) or dietary restriction (DR) feeding regimens. Activity was measured using metabolic Promethion cages over 168 hours (n = 6 mice per group). **B**) Daily food intake (grams) of mice on *ad libitum* (AL) or dietary restriction (DR) feeding regimens. Data represent the mean ± SEM (n = 6 mice/group). *Right*, Total water intake (grams) over one week for mice on AL or DR feeding regimens. Data represent the mean ± SEM (n = 6 mice per group). Statistical significance was calculated via two-tailed, unpaired Student’s t-test. **C**) Change in body weight (grams) over time (hours) for mice on AL or DR conditions. Data represent the mean ± SEM (n = 6 mice per group). **D-G**) Mice were fed ad libitum (AL; black) or restricted to 30%, 40%, or 50% of AL intake (light- to dark-blue) for two weeks. Percent change from baseline in (**D**) total body weight, **E**) Mouse weight change across diets (AL, 30%, 40%, and 50% DR) over 32 days. Data represent mean ± SEM, (**F**) fat mass, and (**G**) lean mass was measured by EchoMRI. Each circle represents an individual mouse; bars indicate mean ± SEM. Statistical significance was calculated via one-way ANOVA multiple comparisons test (n = 6 (30% DR), 8 (40% DR), 8 (50% DR), 6 (AL). **H**) Kaplan–Meier curves of tumor onset in mice fed ad libitum (AL; black) or restricted to 30%, 40% or 50% of AL intake (light to dark blue). The y-axis shows the percentage of tumor-free animals, and the x-axis is days post tumor injection (PTI). Each step down represents an individual mouse developing a tumor equal to or greater than 250mm^3^. Statistical significance was calculated with log rank test with Bonferroni correction (n = 26 (AL), 5 (30% DR), 8 (40%), and 24 (50% DR). *P < 0.05, **P < 0.01, ***P < 0.001, ****P < 0.0001. **Exact P-values are as follows:** Extended Data Fig. 3B, left, *P* < 0.0001, right, *P* < 0.0001; Extended Data Fig. 3D, 30% DR vs. 40% DR, *P* = 0.1447; 30% DR vs. 50% DR, *P* = 0.0025; 30% DR vs. AL, *P* = 0.0322; 40% DR vs. 50% DR, *P* = 0.2321; 40% DR vs. AL, *P* < 0.0001; 50% DR vs. AL, *P* < 0.0001; Extended Data Fig. 3F**:** 30% DR vs. 40% DR, *P* = 0.0454; 30% DR vs. 50% DR, *P* = 0.0011; 30% DR vs. AL, *P* = 0.7572; 40% DR vs. 50% DR, *P* = 0.3543; 40% DR vs. AL, *P* = 0.0039; 50% DR vs. AL, *P* < 0.0001; Extended Data Fig. 3G**:** 30% DR vs. 40% DR, *P* = 0.2651; 30% DR vs. 50% DR, *P* = 0.3589; 30% DR vs. AL, *P* = 0.0538; 40% DR vs. 50% DR, *P* = 0.9963; 40% DR vs. AL, *P* = 0.0004; 50% DR vs. AL, *P* = 0.0006. Extended Data Fig. 3H, shown in figure. Source data

We next evaluated mechanisms underlying the effects of DR on CD8^+^ T cell effector function. Animals on the DR regimen displayed normal levels of physical activity (Extended Data Fig. 3a) but consumed 50% less food and water on average (Extended Data Fig. 3b), resulting in weight loss that plateaued after 3–4 days (Extended Data Fig. 3c). EchoMRI analysis of mice subjected to various levels of DR (that is, 30%, 40% or 50% DR) for 2 weeks revealed greater body-weight loss that plateaued by day 4 (Extended Data Fig. 3c,d). Although 50% DR elicited the largest mean weight reduction, it was not significantly greater than that seen with 40% DR (Extended Data Fig. 3e). Importantly, weight loss under DR was driven almost entirely by fat-mass depletion—with >50% loss in mice on 50% DR—while lean mass loss was similar between the 40% and 50% DR groups (Extended Data Fig. 3f,g). Consistent with these effects on host metabolism, tumour onset was delayed under all DR regimens, with the greatest and most consistent protection seen with 50% DR (Extended Data Fig. 3h).

**Extended Data Fig. 4:**
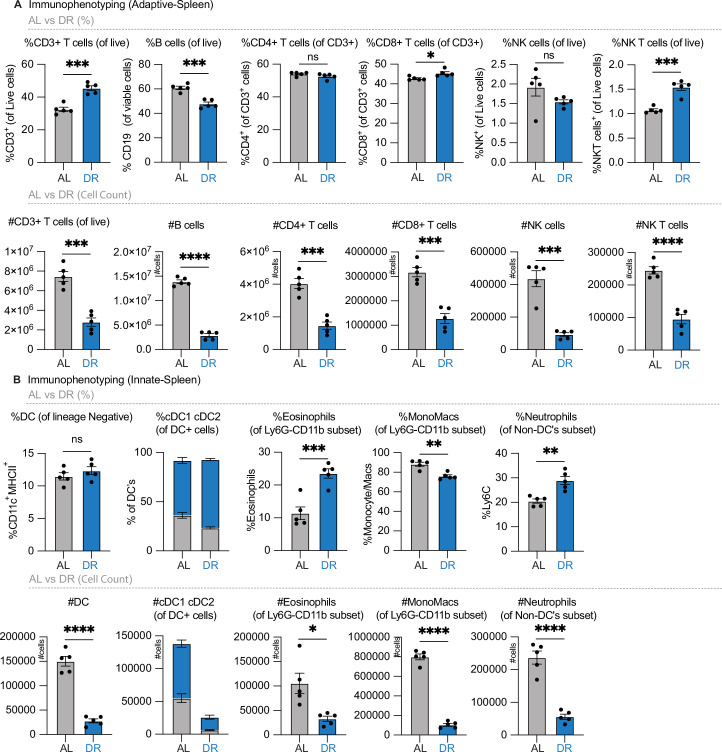
Immunophenotyping of adaptive and innate immune cells under DR. **A**) Adaptive–spleen immunophenotyping. Top panel shows the frequency of major adaptive subsets in spleen from mice fed ad libitum (AL, gray) or on dietary restriction (DR, blue) for two weeks. Frequencies are plotted as percentage of live cells (% CD3⁺ T cells; % CD19⁺ B cells) or as percentage of the parent T-cell pool (% CD4⁺ and % CD8⁺ of CD3⁺) and innate lymphoid subsets (% NK and % NKT of live). Bottom panel shows the corresponding absolute cell counts recovered per spleen. In all plots, each point is one mouse (n = 5 per group); bars indicate mean ± SEM. Statistical significance was calculated via two-tailed, unpaired Student’s t-test. **B**) Innate–spleen immunophenotyping. Top panel show frequencies of myeloid populations: % CD11c⁺ MHCII⁺ dendritic cells (of lineage⁻), the cDC1 and cDC2 subsets (of total DCs), % eosinophils and % monocytes/macrophages (gated on Ly6G⁻ CD11b⁺), and % neutrophils (of non-DC lineage). Bottom panel shows absolute numbers of each innate subset per spleen. In all plots, each point is one mouse (n = 5 per group); bars indicate mean ± SEM. Statistical significance was calculated via two-tailed, unpaired Student’s t-test. *P < 0.05, **P < 0.01, ***P < 0.001, ****P < 0.0001. **Exact P-values are as follows:** Extended Data Fig. 4A, first row (left to right), *P* = 0.0002, *P* = 0.0002, *P* = 0.1477, *P* = 0.0135, *P* = 0.1487, *P* = 0.0002; second row (left to right), *P* = 0.0001, *P* < 0.0001, *P* = 0.0001, *P* = 0.0001, *P* = 0.0001, *P* < 0.0001. Extended Data Fig. 4B, first row (left to right), *P* = 0.3245, *P* = 0.0009, *P* = 0.0011, *P* = 0.0023; second row, *P* < 0.0001, *P* = 0.0101, *P* < 0.0001, *P* < 0.0001. Source data

**Extended Data Fig. 5:**
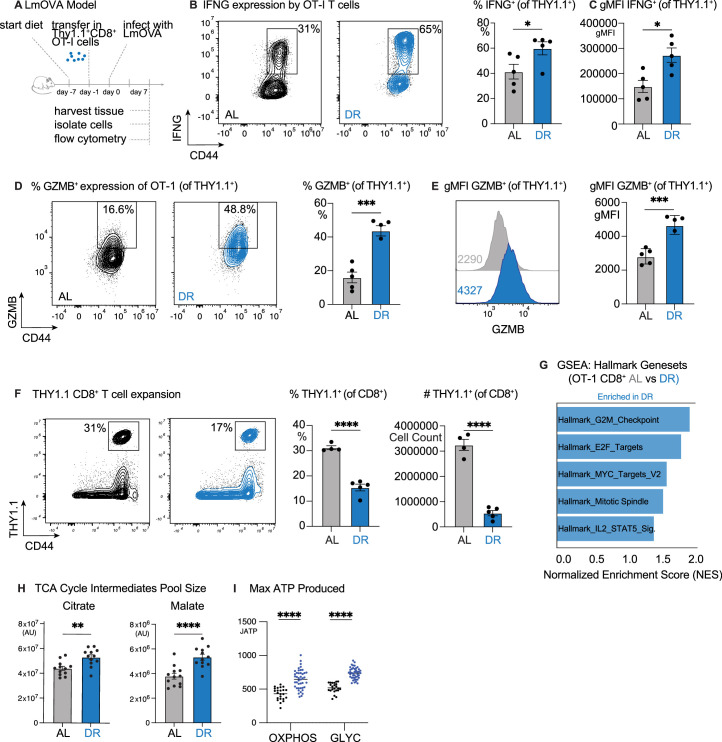
Dietary restriction augments IFN-γ/Granzyme B production and mitochondrial metabolism in OT-I CD8⁺ T cells. **A**) Schematic depicting the experimental setup of adoptive OT-I T cell transfer coupled with attenuated *Lm*OVA infection. Mice were started on AL or DR diets six days before OT-I adoptive transfer. On day 0, mice were injected with LmOVA via tail vein. Seven days post-infection (dpi), spleens were harvested for downstream analyses, including effector function, cell expansion, and metabolic function. **B**) Representative flow cytometry plots showing IFN-γ production and CD44 expression in splenic OT-I CD8⁺ T cells (CD8⁺THY1.1⁺) under AL or DR conditions at 7 dpi. *Right*, bar plot showing the percentage of IFN-γ⁺ cells. Data represent the mean ± SEM (n = 5 mice per group). Statistical significance was calculated via two-tailed, unpaired Student’s t-test. **C**) Bar plot showing the geometric mean fluorescence intensity (gMFI) of IFN-γ in CD8⁺THY1.1⁺ T cells under AL or DR conditions at 7 dpi. Data represent the mean ± SEM (n = 5 mice per group). Statistical significance was calculated via two-tailed, unpaired Student’s t-test. **D**) Representative flow cytometry plots showing GZMB production and CD44 expression in splenic OT-I CD8⁺ T cells (CD8⁺THY1.1⁺) under AL or DR conditions at 7 dpi. *Right*, bar plot showing the percentage of GZMB^+^ cells. Data represent the mean ± SEM (n = 5 mice per group). Statistical significance was calculated via two-tailed, unpaired Student’s t-test. **E**) Representative histogram of GZMB^+^ (of CD8^+^THY1.1^+^) T cells comparing AL and DR conditions. *Right*, bar plot showing the geometric mean fluorescence intensity (gMFI) of IFN-γ in CD8⁺THY1.1⁺ T cells under AL or DR conditions at 7 dpi. Data represent the mean ± SEM (n = 5 mice per group). Statistical significance was calculated via two-tailed, unpaired Student’s t-test. **F**) Representative flow cytometry plots showing expansion of splenic OT-I CD8⁺ T cells (THY1.1 versus CD44) 7 dpi. *Right*, Bar plots showing the percentage and absolute number of THY1.1⁺ antigen-specific CD8⁺ T cells expanded under AL or DR conditions at 7 dpi. Data represent the mean ± SEM (n = 4 AL and 5 DR micegroup). Statistical significance was calculated via two-tailed, unpaired Student’s t-test. **G**) Shown are the top significantly enriched pathways (false discovery rate [FDR] < 0.05) from the Hallmark gene set collection (MSigDB “H”) in the DR versus AL comparison from the proteomic data collected in (I). Proteins were mapped to their corresponding genes, and a ranked list (for example, by fold change) was analyzed using fgsea. Bars indicate the normalized enrichment score (NES), with positive NES reflecting enrichment in the DR condition. **H**) Bar plots showing the pool sizes of TCA cycle intermediates citrate and malate in antigen-specific CD8⁺THY1.1⁺ T cells from LmOVA-infected mice (7 dpi) under AL or DR conditions. Data represent the mean ± SEM (n = 12 mice per group). Statistical significance was calculated via two-tailed, unpaired Student’s t-test. **I**) Maximal ATP production rates (J_ATP) from OXPHOS and GLY for CD8⁺THY1.1⁺ T cells under AL or DR conditions. Data represent the mean ± SD (n = 23 AL and 44 DR technical replicates). Statistical significance was calculated via one-way ANOVA multiple comparisons test. **Exact P-values are as follows:** Extended Data Fig. 5B, P = 0.045; Extended Data Fig. 5C, P = 0.0103; Extended Data Fig. 5D, P = 0.0002; Extended Data Fig. 5E, P = 0.0004; Extended Data Fig. 5F, left, *P* < 0.0001, right, *P* < 0.0001; Extended Data Fig. 5H, left, *P* = 0.0021, right, *P* < 0.0001; Extended Data Fig. 5I, left, *P* < 0.0001, right, *P* < 0.0001. *P < 0.05, **P < 0.01, ***P < 0.001, ****P < 0.0001. Source data

Baseline immune profiling revealed an overall decrease in immune cell numbers in the spleens of DR-fed mice, although the proportion of CD4^+^ and CD8^+^ T cell populations between diets was not dramatically altered (Extended Data Fig. 4a,b and Supplementary Table 3), consistent with previous reports^38,40^. To control for systemic effects of DR on CD8^+^ T cell homoeostasis, we used a T cell adoptive transfer model coupled to *Listeria monocytogenes* (*Lm*) infection, which elicits robust CD8^+^ T_eff_ cell responses in vivo^21,43,44^. Thy1.1^+^ OT-I T cell receptor transgenic CD8^+^ T cells were transferred into AL or DR-fed mice, followed by infection with attenuated *Lm-*expressing ovalbumin (*Lm*OVA), and splenocytes extracted 7 days post infection (dpi) for ex vivo functional analysis (Extended Data Fig. 5a). Similar to CD8^+^ TIL from DR-fed mice, OT-I T cells responding to *Lm*OVA infection under DR-fed conditions displayed increased effector function when analysed ex vivo, characterized by an increase in the percentage of IFNγ-producing T cells (Extended Data Fig. 5b), higher IFNγ production on a per-cell basis (Extended Data Fig. 5c) and an increased percentage of GZMB^+^ CD8^+^ T_eff_ cells (Extended Data Fig. 5d). Elevated GZMB protein levels displayed by DR-conditioned OT-I T cells (Extended Data Fig. 5e) was consistent with the elevated GZMB production displayed by CD8^+^ TIL under DR (Fig. 2b). Ex vivo OT-I CD8^+^ T cells displayed increased effector function in DR-fed mice despite an overall reduction in expansion following *Lm*OVA infection (Extended Data Fig. 5f and Supplementary Table 4).

To identify mechanisms by which DR enhances CD8^+^ T_eff_ cell function, we analysed changes in global protein levels in OT-I T cells isolated from AL-fed or DR-fed mice following *Lm*OVA infection (as in Extended Data Fig. 5a). At 7 dpi, OT-I T_eff_ cells isolated from DR-conditioned mice displayed enrichment of a distinct set of 277 proteins associated with anabolic growth and metabolism (that is, G2M checkpoint, E2F and MYC targets, interleukin-2 (IL-2) signalling) (Fig. 2d, Extended Data Fig. 5g and Supplementary Table 4). Using the fold-change data from Fig. 2d (with proteins first mapped to gene symbols) and the results in Supplementary Table 4, we performed gene set enrichment analysis (GSEA) against the Hallmark, C2 and C5 gene collections. This analysis revealed significant enrichment of the tricarboxylic acid (TCA) cycle pathway (Fig. 2e and Supplementary Table 4), which underpins both bioenergetic and biosynthetic functions in CD8⁺ T cells^11^. Consistent with this observation, OT-I T_eff_ cells isolated from DR-conditioned mice responding to *Lm*OVA infection displayed increased levels of TCA cycle metabolites compared to control-fed animals (Fig. 2f and Extended Data Fig. 5h). Seahorse analysis of OT-I T_eff_ cells isolated from *Lm*OVA-infected mice revealed an increase in both the extracellular acidification rate (a measure of glycolysis) and oxygen consumption rate (a measure of OXPHOS) in DR-conditioned T_eff_ cells compared to AL-fed controls (Fig. 2g,h). This corresponded to significant increases in both oxidative and glycolytic ATP production in T_eff_ cells from DR-conditioned mice compared to AL-fed controls (Fig. 2i) and almost a doubling in maximal ATP production capacity (Extended Data Fig. 5i). Moreover, OT-I T_eff_ cells responding to *Lm*OVA infection displayed increased mitochondrial membrane potential (as determined by TMRM staining) under DR conditions compared to AL-fed controls (Fig. 2j). Collectively, these data indicate that restricting food intake (by DR) enhances CD8^+^ T_eff_ cell metabolic fitness through effects on the TCA cycle and OXPHOS.

### DR antagonizes terminal CD8^+^ T cell exhaustion in the TME

**Fig. 3:**
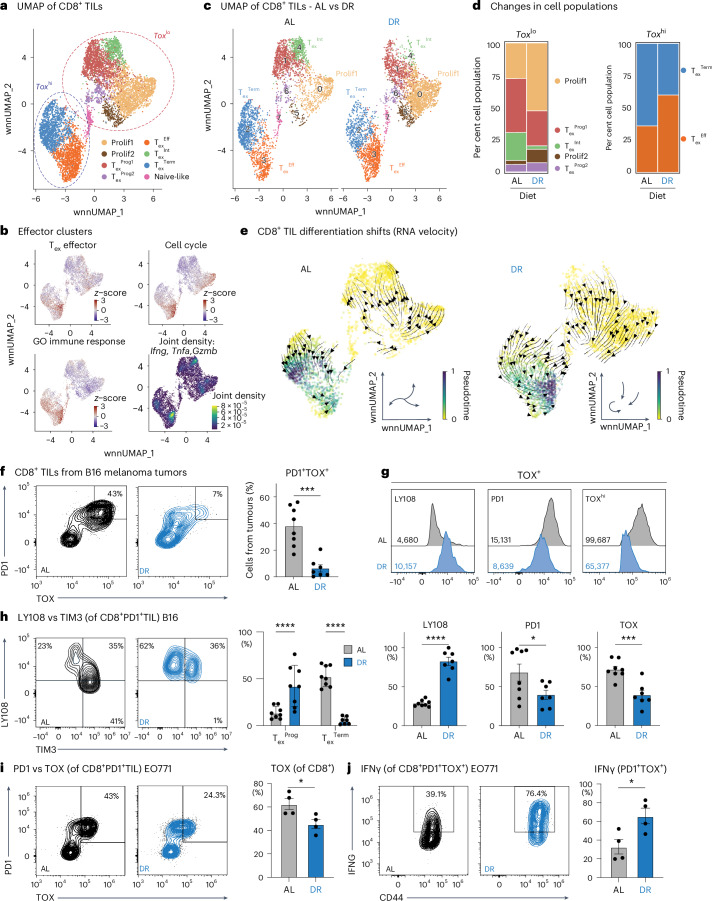
DR antagonizes terminal CD8^+^ T cell exhaustion in the TME. **a**, WnnUMAP of 7,005 activated (CD44^+^) CD8⁺ TILs from B16 melanoma tumours (combined AL and DR) (*n* = 4). Legend indicates unique T cell clusters called based on RNA expression and ADTs. T cell clusters with high and low *Tox* expression are highlighted by circles. **b**, Overlay of wnnUMAP for activated CD8^+^ TIL from **a** and MSigDB gene expression signatures for T_ex_^Eff^, cell cycle and Gene Ontology (GO) Immune Response pathways for TILs isolated from B16 tumours (combined AL and DR). Joint density plot indicates the highest combined expression/cell of *Ifng*, *Tnfa* and *Gzmb* among activated CD8⁺ T cells from B16 tumours. **c**, wnnUMAP of activated CD8⁺ TILs divided by dietary conditions (AL, 3,348 cells; DR, 3,657 cells). Prominent CD8⁺ T cell clusters are indicated. **d**, Stacked bar graphs showing the percentage of CD8⁺ T cell populations divided by *Tox* expression (*Tox*^lo^ versus *Tox*^hi^). **e**, RNA velocity plots inferring cellular differentiation trajectory for CD8⁺ T cells infiltrating B16 tumours from AL-fed or DR-fed mice. Trajectories were derived from the dynamical prediction model scVelo, with representative directionality for T cells under each diet shown in the inset. **f**, PD1 and TOX expression in B16 tumour-infiltrating CD8⁺ T cells isolated from AL or DR mice 14 days PTI. Left, representative flow cytometry plots for PD1 versus TOX expression; right, bar graph showing the percentage of PD1⁺TOX⁺ CD8⁺ T cells isolated from tumours. Data represent the means; error bars, s.e.m. (*n* = 8 AL and 7 DR mice). Statistical significance was calculated with a two-tailed, unpaired Student’s *t*-test. **g**, Expression of stemness and exhaustion markers in TOX⁺ CD8⁺ T cells isolated from B16 tumours from AL-fed or DR-fed mice. Top, representative histograms of LY108, PD1 and TOX expression. Geometric mean fluorescence intensity averaged across all biological replicates is indicated. Bottom, bar graphs quantifying the percentage of CD8^+^ cells expressing each of the indicated proteins. Data represent the means; error bars, s.e.m. (*n* = 8 AL and 7 DR mice). Statistical significance was calculated with a two-tailed, unpaired Student’s *t*-test. **h**, Expression of LY108 versus TIM3 among PD1⁺ CD8⁺ TILs from B16 tumours. Left, representative flow cytometry plots showing LY108 and TIM3 expression; right, bar graph showing percentages of progenitor (T_ex_^Prog^, LY108^+^TIM3^−^) and terminal (T_ex_^Term^, LY108^−^TIM3^+^) T_ex_ cell subsets under AL and DR conditions. Data represent the means; error bars, s.e.m. (*n* = 8 mice per group). Statistical significance was calculated with a two-way ANOVA multiple comparisons test. **i**, PD1 and TOX expression in EO771 (breast cancer) tumour-infiltrating CD8⁺ T cells isolated from AL or DR mice 14 days PTI. Left, representative flow cytometry plots for PD1 versus TOX expression; right, bar graph showing the percentage of TOX⁺ CD8⁺ T cells isolated from tumours. Data represent the means; error bars, s.e.m. (*n* = 4 mice per group). Statistical significance was calculated with a two-tailed, unpaired Student’s *t*-test. **j**, CD44 and IFNγ expression in EO771 (breast cancer) tumour-infiltrating CD8⁺ T cells isolated from AL or DR mice 14 days PTI. Left, representative flow cytometry plots for CD44 versus IFNγ expression; right, bar graph showing the percentage of IFNγ-producing cells from PD1^+^TOX⁺ CD8⁺ T cells isolated from EO771 tumours. Data represent the means; error bars, s.e.m. (*n* = 4 mice per group). Statistical significance was calculated with a two-tailed, unpaired Student’s *t*-test. *P < 0.05; **P < 0.01; ***P < 0.001; ****P < 0.0001. Exact *P* values are as follows: **f**, *P* = 0.0002; **g**, left, *P* < 0.0001, middle, *P* = 0.0469, right, *P* = 0.0004; **h**, left, *P* = 0.0003, right, *P* < 0.0001; **i**, *P* = 0.032; **j**, *P* = 0.0275. Source data

**Extended Data Fig. 6:**
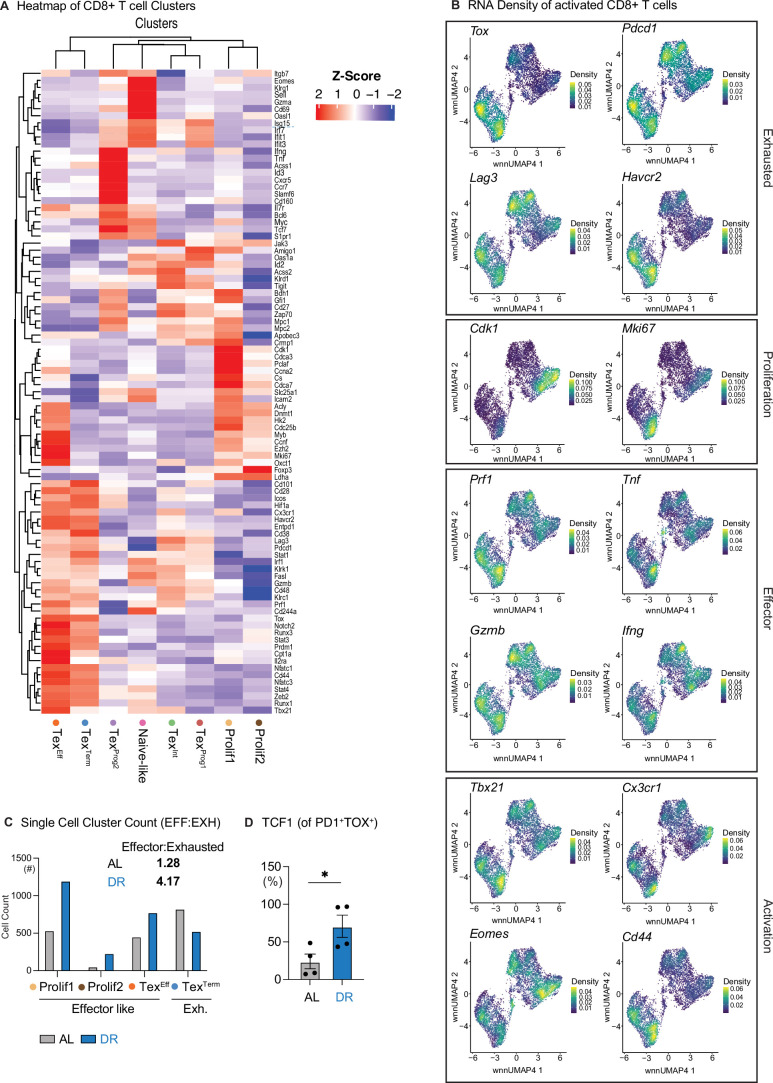
Dietary restriction alters the CD8^+^ T cell tumour microenvironment. **A**) Heatmap showing Log_2_-normalized expression of key genes highlighting population differences among cell clusters in TILs isolated from B16 tumors (combined AL and DR conditions). Expression shown as Z-score. **B**) RNA density plots of activated CD8⁺ T cell clusters represented on a wnnUMAP, displaying the density of genes associated with exhaustion, proliferation, effector function, and activation gene sets (joint plot of AL and DR conditions). **C**) Bar plot depicting cell counts from CITE-sequencing experiments for Prolif1, Prolif2, TexEff, and TexTerm CD8⁺ TIL clusters under AL and DR conditions. The Effector:Exhausted ratio is displayed based on cell counts. **D**) Bar plot showing the percent expression of TCF1 of PD1^+^TOX^+^CD8^+^ TILs (EO771) between AL and DR conditions. Data represent the mean ± SEM (n = 4 mice/group). Statistical significance was calculated via two-tailed, unpaired Student’s t-test. *P < 0.05, **P < 0.01, ***P < 0.001, ****P < 0.0001. **Exact P-values are as follows:** Extended Data Fig. 6D, P = 0.0382. Source data

Our findings indicate that DR increases the proportion of functional CD8^+^ T_eff_ cells within the TME. Therefore, we used our CITE-seq datasets to characterize how DR impacts CD8^+^ T cell subsets within TIL populations. Cell clusters were classified based on a combination of gene features, gene density, antibody-derived tags (ADTs) and MSigDB pathway analysis (Extended Data Fig. 6a,b and Supplementary Table 5). Cellular indexing of both protein epitopes and transcriptional profiling revealed two major CD8^+^ TIL populations in B16 tumours based on gene expression for *Tox*, a key transcriptional regulator of T cell exhaustion^7,8^ (Fig. 3a). The accumulation of T_ex_ cells in tumours is driven by TOX and is reinforced through epigenetic programming^7–10^, ultimately limiting the ability of CD8^+^ T cells to control tumour growth. In B16 tumours, the *Tox*^lo^ population consisted of five main subclusters, including two *Mki67*^+^ proliferating T_eff_ cell populations (Prolif1 and Prolif2), two progenitor T_ex_ cell populations (T_ex_^Prog1^, T_ex_^Prog2^), an intermediate exhausted (T_ex_^Int^) population and a small naive-like cell population (Fig. 3a and Extended Data Fig. 6a,b). *Tox*^hi^ CD8^+^ T cells displayed elevated transcript levels of inhibitory receptor genes (*Lag3*^+^*Havcr2*^+^*Pcdc1*^+^) but clustered into two distinct groups: a conventional terminally exhausted (T_ex_^Term^) population and a proliferating (*Mki67*^+^) effector-like exhausted population (T_ex_^Eff^) (Fig. 3a,b and Extended Data Fig. 6a,b)^5^. Fast gene set enrichment analysis (fgsea) identified the T_ex_^Eff^ cluster as having the most effector-like properties, including enrichment for immune response and cell cycle genes (Fig. 3b). T_ex_^Eff^ cells also displayed increased expression of genes encoding effector molecules (that is, *Ifng*, *Tnfa* and *Gzmb*) (Fig. 3b), despite elevated expression of transcripts encoding *Tox* and inhibitory receptors (that is, *Pdcd1*, *Lag3*, *Havcr2*) (Extended Data Fig. 6b).

Single-cell hashtagging strategies allowed us to track CD8^+^ TIL populations in B16 tumours from either AL-fed or DR-fed mice. Analysis of CD8^+^ TIL populations revealed two major trends induced by DR feeding. First, for the *Tox*^lo^ CD8^+^ TIL subset, we observed an expansion in proliferating T_eff_ cell populations (Prolif1, Prolif2) and a decrease in T_ex_^Int^ cells in animals conditioned to DR (Fig. 3c,d). Second, the type of *Tox*-expressing T_ex_ cells found in tumours was highly dependent on diet. Tumours from AL-fed mice contained more T_ex_^Term^ CD8^+^ T cells, while tumours from DR mice contained a greater proportion of T_ex_^Eff^ (Fig. 3c,d). To determine whether the diet-induced changes in T_ex_ cell populations reflect differences in CD8^+^ T cell differentiation, we used RNA velocity analysis, a computational method that infers cellular differentiation trajectories based on the relative abundances of spliced and unspliced transcripts in single cells^45^. Under AL conditions, RNA velocity inference showed a directional flow away from the proliferating effector population towards T_ex_^Int^ and ultimately T_ex_^Term^ populations (Fig. 3e). By contrast, under DR, the direction of CD8^+^ T cell trajectory culminated in the effector-like T_ex_^Eff^ population (Fig. 3e). This change in differentiation trajectory corresponded to a major shift in the ratio of effector to terminally exhausted T cell populations in B16 tumours, moving from an equal ratio of effector (proliferating and T_ex_^Eff^) to T_ex_^Term^ cells under AL-fed conditions to a 4:1 ratio under DR (Extended Data Fig. 6c). Collectively, these results suggest that DR alters CD8^+^ TIL fate, shifting CD8^+^ T cells away from terminal exhaustion and towards a more effector-like state that favours tumour control.

We next used flow cytometry to characterize the impact of DR on CD8^+^ T cell function within the TME. Consistent with our CITE-seq analysis, the number of CD8^+^ T_ex_^Term^ cells—as determined by reduced TOX and inhibitory receptor (PD1^+^TOX^+^) expression—decreased significantly in tumours from DR-conditioned animals compared to AL-fed mice 14 days post tumour implantation (Fig. 3f). TOX-expressing CD8^+^ TILs from DR-fed mice displayed increased expression of stemness markers (LY108/SLAMF6) while showing reduced features of terminal exhaustion (that is, lower PD1 and TOX expression) compared to AL-fed controls (Fig. 3g). Using conventional markers for terminally exhausted (LY108^−^TIM3^+^) versus progenitor exhausted (LY108^+^TIM3^−^) cells^4^, the majority of PD1^+^ CD8^+^ TILs in B16 tumours from DR-fed mice were progenitor exhausted or an intermediate phenotype (LY108^+^TIM3^+^) (Fig. 3h). To validate our observations in an independent tumour model, we analysed TILs from EO771 breast cancer tumours and found a similar reduction in PD1^+^TOX^+^ CD8^+^ T cells within tumours from DR-conditioned mice compared to AL-fed controls (Fig. 3i). Indeed, TOX-expressing CD8^+^ TILs from E0771 tumours grown in DR-fed mice exhibited increased IFNγ production (Fig. 3j) and elevated TCF1 levels, a marker of cell stemness (Extended Data Fig. 6d). These findings indicate that DR enhances both the frequency and functional capacity of effector CD8⁺ T cells, promoting a less exhausted, more stem-like phenotype across tumour models.

### DR enhances CD8^+^ T cell metabolic fitness via ketone body metabolism

**Fig. 4:**
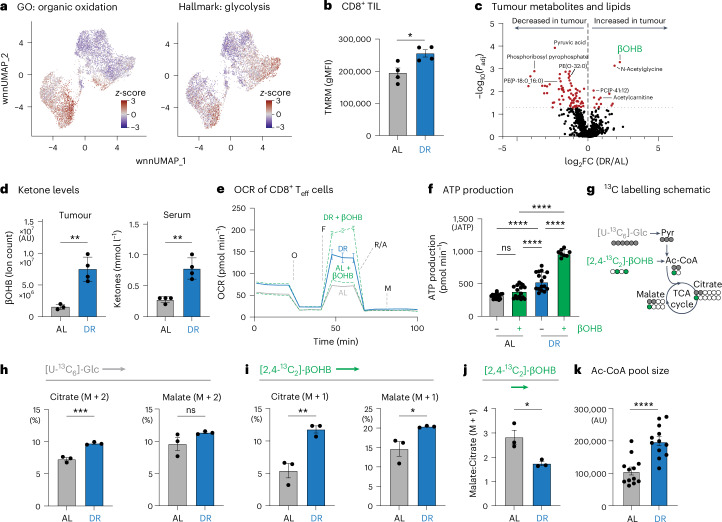
DR enhances CD8^+^ T cell metabolic fitness via ketone body metabolism. **a**, Overlay of wnnUMAP for 7,005 activated (CD44^+^) CD8⁺ TIL from B16 melanoma tumours (both AL and DR) and MSigDB gene expression signatures for organic oxidation and glycolysis pathways. **b**, Mitochondrial membrane potential of AL-conditioned and DR-conditioned CD8⁺ T cells. TMRM staining (gMFI) of CD8^+^ OT-I TIL isolated from B16 tumours from AL-fed or DR-fed mice (14 days PTI). Data represent the means; error bars, s.e.m. (*n* = 4 mice per group). Statistical significance was calculated with a two-tailed, unpaired Student’s *t*-test. **c**, Volcano plot showing the log_2_(fold change) in metabolite and lipid abundance in B16 tumours isolated from AL versus DR mice (*n* = 3–4 mice per group). Select metabolites enriched in AL and DR tumours are annotated. Statistical significance was determined using a −log_10_(*P* value) of 1.30 as a cutoff value for data visualization. **d**, Ketone body levels in the serum and tumours of AL-fed versus DR-fed mice after 21 days on diet (14 days of tumour growth). Left, βOHB abundance in B16 tumours from AL-fed versus DR-fed mice as quantified by mass spectrometry; right, ketone body abundance in serum as quantified by enzyme assay. Data represent the means; error bars, s.e.m. (left, *n* = 3 AL and 4 DR mice; right, *n* = 4 mice per group). Statistical significance was calculated with a two-tailed, unpaired Student’s *t*-test. **e**,**f**, Bioenergetic profile of AL-conditioned and DR-conditioned CD8⁺ T cells exposed to βOHB. **e**, OCR plot for antigen-specific CD8⁺ T cells isolated from *Lm*OVA-infected AL-fed or DR-fed mice (7 dpi). T cells were cultured with or without 1.5 mM βOHB 60 min before the start of the assay. **f**, Maximum ATP production rates from OXPHOS for CD8⁺ T cells from **e**. Data represent the means; error bars, s.d. (*n* = 16 AL, 16 AL + βOHB, 16 DR and 8 DR + βOHB, technical replicates). Statistical significance was calculated with one-way ANOVA with multiple comparisons. **g**, Schematic depicting ^13^C labelling patterns in acetyl-CoA (Ac-CoA) and TCA cycle intermediates from U-[^13^C_6_]-glucose and [2,4-^13^C_2_]-βOHB. **h**–**j**, ^13^C labelling of TCA cycle intermediates in AL-conditioned versus DR-conditioned CD8⁺ T cells. CD8⁺ T cells isolated from *Lm*OVA-infected AL-fed or DR-fed mice (7 dpi) were cultured for 2 h ex vivo in VIM medium containing 5 mM U-[^13^C_6_]-glucose and 1.5 mM [2,4-^13^C_2_]-βOHB. Shown is the per cent incorporation of U-[^13^C_6_]-glucose-derived carbon (M + 2) (**h**) and [2,4-^13^C_2_]-βOHB-derived carbon (M + 1) (**i**) into citrate and malate. **j**, Ratio of [2,4-^13^C_2_]-βOHB-labelled malate (M + 1) to citrate (M + 1) for CD8^+^ T cells. Data represent the means; error bars, s.e.m. (*n* = 3 mice per group). Statistical significance was calculated with a two-tailed, unpaired Student’s *t*-test. **k**, Bar graph of Ac-CoA abundance in CD8⁺ T cells isolated from *Lm*OVA-infected AL-fed or DR-fed mice (7 dpi). AU, arbitrary units. Data represent the means; error bars, s.e.m. (*n* = 12 mice per group). Statistical significance was calculated with a two-tailed, unpaired Student’s *t*-test. *P < 0.05; **P < 0.01; ***P < 0.001; ****P < 0.0001. Exact *P* values are as follows: **b**, *P* = 0.0194; **d**, left, *P* = 0.0035, right, *P* = 0.0014; **f**, ns = 0.1985, ****P < 0.0001; **h**, left, *P* = 0.0011, right, *P* = 0.158; **i**, left, *P* = 0.0068; rightinfected AL-fed or DR-fed, *P* = 0.0381; **j**, *P* = 0.0244; **k**, *P* < 0.0001. Source data

CD8⁺ T cells rely on glycolysis and OXPHOS to fuel their proliferation and effector function^12–14^, but are capable of metabolizing a diverse set of metabolic substrates to fuel these processes^21,46,47^. Given the impact of DR on CD8^+^ T cell fate in the TME, we assessed whether DR alters the metabolic programming of CD8^+^ T cells needed for tumour control. We first conducted metabolism-focused GSEA on our CITE-seq datasets to define the metabolic features of CD8^+^ TIL subsets. This analysis revealed two distinct metabolically active CD8^+^ TIL populations in B16 tumours. CD8^+^ T cells with effector properties (Prolif1, T_ex_^Eff^) displayed the highest glycolytic signature (Fig. 4a), consistent with their high proliferative rate. Notably, T_ex_^Eff^ cells—which preferentially expand in tumours under DR (Fig. 3a,b)—displayed the highest OXPHOS signature of all CD8^+^ TIL subsets (Fig. 4a). Moreover, like DR-conditioned T_eff_ cells responding to *Listeria* infection (Fig. 2j), CD8^+^ TILs isolated from B16 tumours displayed increased TMRM staining (mitochondrial membrane potential) under DR conditions compared to CD8^+^ TILs from AL-fed controls (Fig. 4b). Therefore, the most metabolically active CD8^+^ TIL subsets—by gene expression signatures and mitochondrial membrane potential—are the effector cell populations enhanced by DR treatment.

**Extended Data Fig. 7:**
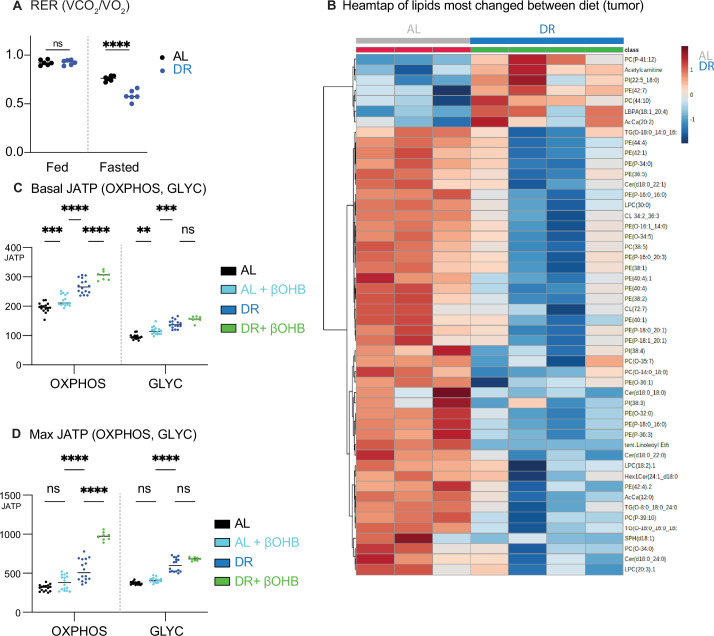
Dietary restriction enhances metabolic function of CD8^+^ T cells. **A**) Mean respiratory exchange ratio (RER; VCO_2_/VO_2_) of mice on AL or DR feeding regimens measured using Promethion metabolic cages. “Fed” represents the average peak RER post-feeding, while “Fasted” represents the lowest RER during fasting periods. Data represent the mean ± SEM (n = 6 mice per group). Statistical significance was calculated via two-way ANOVA multiple comparisons test. **B**) Heatmap showing the most significant lipid changes (Log_2_ fold change) in tumors isolated from mice on AL or DR diets 14 days post tumor implantation (n = 3–4 mice per group). **C**) Theoretical basal ATP production rates (J_ATP) from OXPHOS and GLY in CD8⁺ T cells cultured with or without β-hydroxybutyrate (βOHB) under AL or DR conditions. Data represent the mean ± SEM (n = 16 AL, 16 AL + βOHB, 16 DR, and 8 DR + βOHB, technical replicates). Statistical significance was calculated via two-way ANOVA multiple comparisons test. **D**) Theoretical maximal ATP production rates (J_ATP) from OXPHOS and GLY under the same conditions as in (C). Data represent the mean ± SEM (n = 16 AL, 16 AL + βOHB, 16 DR, and 8 DR + βOHB, technical replicates). Statistical significance was calculated via two-way ANOVA multiple comparisons test. *P < 0.05, **P < 0.01, ***P < 0.001, ****P < 0.0001. **Exact P-values are as follows:** Extended Data Fig. 7A, ns = 0.473, *P* < 0.0001; Extended Data Fig. 7C (Image A): *OXY* — AL vs. AL + βHB, *P* = 0.0004; AL vs. DR, *P* < 0.0001; AL vs. DR + βHB, *P* < 0.0001; AL + βHB vs. DR, *P* < 0.0001; AL + βHB vs. DR + βHB, *P* < 0.0001; DR vs. DR + βHB, *P* < 0.0001. *GLY* — AL vs. AL + βHB, *P* = 0.0034; AL vs. DR, *P* < 0.0001; AL vs. DR + βHB, *P* < 0.0001; AL + βHB vs. DR, *P* = 0.0008; AL + βHB vs. DR + βHB, *P* < 0.0001; DR vs. DR + βHB, ns = 0.1. Extended Data Fig. 7D (Image B): *OXY* — AL vs. AL + βHB, ns = 0.0658; AL vs. DR, *P* < 0.0001; AL vs. DR + βHB, *P* < 0.0001; AL + βHB vs. DR, *P* < 0.0001; AL + βHB vs. DR + βHB, *P* < 0.0001; DR vs. DR + βHB, *P* < 0.0001. *GLY* — AL vs. AL + βHB, ns = 0.5079; AL vs. DR, *P* < 0.0001; AL vs. DR + βHB, *P* < 0.0001; AL + βHB vs. DR, *P* < 0.0001; AL + βHB vs. DR + βHB, *P* < 0.0001; DR vs. DR + βHB, ns = 0.1091. Source data

Dietary modifications that alter systemic metabolism in mice can impact nutrient availability in the TME^24,30,48^. Therefore, we reasoned that DR impacts CD8^+^ T cell metabolism, in part, by altering the availability of specific nutrients in vivo. Indirect calorimetry revealed that mice conditioned to DR displayed a significant decrease in their respiratory exchange ratio during the fasted—but not the fed—state (Extended Data Fig. 7a), indicating increased lipid oxidation in DR-fed mice. We next used mass spectrometry to profile diet-induced alterations in lipid and metabolite abundance in B16 tumours. We observed a general decrease in fatty acid and lipid abundance in DR tumours compared to AL-fed controls (Fig. 4c, Extended Data Fig. 7b and Supplementary Tables 6 and 7). This change in tumour lipid abundance is consistent with lipid mobilization from adipose tissue and a decrease in respiratory exchange rate, favouring oxidation triggered by DR^30,38^ (Extended Data Fig. 7a). By contrast, only a small number of metabolites were enriched in tumours from DR-treated mice, including increased abundance of the ketone body βOHB (Fig. 4c). Elevated βOHB levels were observed in both tumours and serum from DR-fed mice compared to AL-fed mice (Fig.4d).

The increase in circulating and intratumoral βOHB levels under DR was notable, given recent evidence linking ketone body metabolism to enhanced T cell effector function^22,49^. Using Seahorse bioenergetic analysis and ^13^C-labelled metabolic tracers, we observed a striking difference in βOHB use by CD8^+^ T_eff_ cells based on diet. First, in ex vivo assays, augmenting DR-conditioned OT-I T_eff_ cells with βOHB further increased their maximal oxygen consumption rate (Fig. 4e). This change corresponded to an approximately fourfold increase in maximal oxidative ATP production capacity compared to CD8^+^ T_eff_ cells from AL-fed animals (Fig. 4f and Extended Data Fig. 7c,d). Next, we cultured OT-I T_eff_ cells from *Lm*OVA-infected mice ex vivo in physiologic medium (VIM)^21^ containing fully labelled [^13^C_6_]-glucose and partially labelled [2,4-^13^C_2_]-βOHB. In this strategy, breakdown of [^13^C_6_]-glucose generates M + 2 labelled TCA cycle intermediates, whereas [2,4-^13^C_2_]-βOHB generates M + 1 labelled intermediates (Fig. 4g), thereby facilitating a direct comparison of the contribution of each fuel type to TCA cycle metabolism^22^. We observed a small but significant increase in [^13^C_6_]-glucose-derived citrate (M + 2) in DR-conditioned CD8^+^ T_eff_ cells (Fig. 4h and Supplementary Table 8); however, citrate labelling from [2,4-^13^C_2_]-βOHB (M + 1) doubled in CD8^+^ T_eff_ cells from DR compared to AL-fed mice (Fig. 4i). DR-conditioned OT-I T_eff_ cells also displayed a lower ratio of [2,4-^13^C_2_]-βOHB-labelled malate-to citrate (Fig. 4j), indicative of increased export of mitochondrial citrate to the cytosol^50^. Consistent with these observations, steady-state acetyl-CoA levels were twofold higher in CD8^+^ T_eff_ cells from DR-fed mice relative to T cells from AL-fed controls (Fig. 4k). Thus, DR increases both systemic βOHB availability and its use by CD8^+^ T cells.

### Chronic T cell stimulation promotes increased βOHB metabolism

**Fig. 5:**
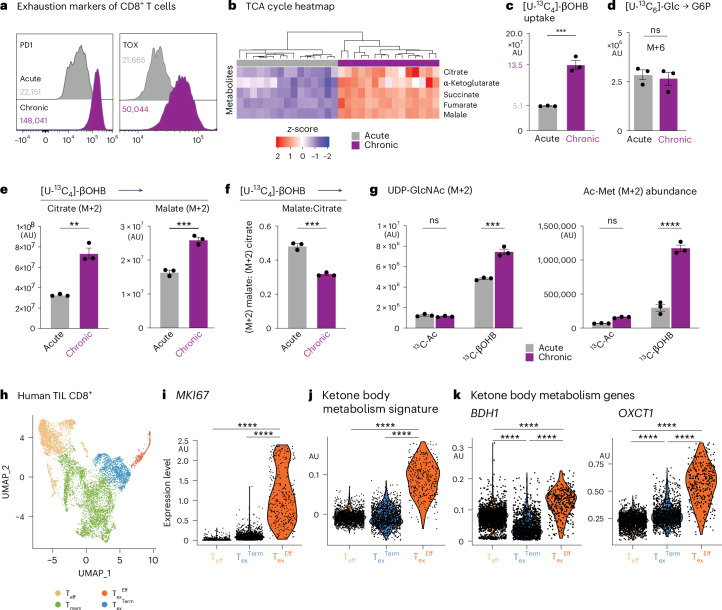
Chronic TCR stimulation promotes increased βOHB metabolism. **a**, Inhibitory receptor expression on in vitro-stimulated T cells. Representative histograms of exhaustion marker expression (PD1, TOX) in CD8⁺ T cells under acute and chronic stimulation conditions. Data represent the means; error bars, s.e.m. (*n* = 3 mice per group). **b**, Heatmap showing the relative abundance (*z*-score) of TCA cycle-derived metabolites from CD8⁺ T cells exposed to acute versus chronic stimulation with anti-CD3 and anti-CD28 antibodies in vitro (*n* = 3 mice per group). **c**, Bar graph depicting total abundance of ^13^C-labelled βOHB in acute versus chronic stimulated CD8⁺ T cells. Data represent the means; error bars, s.e.m. (*n* = 3 mice per group). Statistical significance was calculated with a two-tailed, unpaired Student’s *t*-test. **d**, Bar graph depicting [U-^13^C_6_]-glucose labelling into glucose-6-phosphate (G6P), M + 6, in acute and chronic stimulated CD8^+^ T cells. Statistical significance was calculated with a two-tailed, unpaired Student’s *t*-test. Data represent the means; error bars, s.e.m. (*n* = 3 mice per group). **e**,**f**, ^13^C labelling of TCA cycle intermediates in CD8⁺ T cells exposed to chronic antigen stimulation. CD8⁺ T cells exposed to acute versus chronic stimulation were cultured in VIM medium containing 0.85 mM [^13^C_4_]-βOHB for 2 h. **e**, Total abundance of [^13^C_4_]-βOHB-derived citrate and malate. **f**, Ratio of [^13^C_4_]-βOHB-labelled malate (M + 2) to citrate (M + 2) for T cells from **e**. Data represent the means; error bars, s.e.m. (*n* = 3 mice per group). Statistical significance was calculated with a two-tailed, unpaired Student’s *t*-test. **g**, Bar graph showing [^13^C_2_]-acetate into UDP-GlcNAc (M + 2) in acute and chronically stimulated CD8^+^ T cells, as well as [^13^C_4_]-βOHB into UDP-GlcNAc (M + 2) acute and chronically stimulated CD8^+^ T cells (total abundance). Right, bar graph showing [^13^C_2_]-acetate into Ac-Met (M + 2) in acute and chronically stimulated CD8^+^ T cells as well as [^13^C_4_]-βOHB into Ac-Met (M + 2) acute and chronically stimulated CD8^+^ T cells (total abundance). Data represent the means; error bars, s.e.m. (*n* = 3 mice per group). Statistical significance was calculated via two-way ANOVA with multiple comparisons. **h**, UMAP of 8,552 human CD8⁺ TILs from GSE98638. Shown are unique clusters for effector (T_eff_), memory (T_mem_), terminally exhausted (T_ex_^Term^) and proliferating exhausted (T_ex_^Eff^) CD8⁺ T cell populations. **i**–**k**, Violin plots of gene expression across human CD8⁺ TIL subsets. **i**, Expression of *MKI67* human gene between CD8^+^ T cell subsets. **j**, Expression of ketolysis signature genes (ketolysis gene set: *ACAT1*, *ACAT2*, *BDH1*, *BDH2*, *HMGCL*, *HMGCS1*, *HMGCS2*, *OXCT1*, *OXCT2*). Statistical significance was assessed by one-way ANOVA with Dunnett’s multiple comparisons test and a 5% significance level. **k**, Violin plots of *BDH1* and *OXCT1* gene expression between different human CD8^+^ T cell subsets. *P < 0.05; **P < 0.01; ***P < 0.001; ****P < 0.0001. Exact *P* values are as follows: **c**, *P* = 0.0007; **d**, *P* = 0.7405; **e**, left, *P* = 0.0120, right, *P* = 0.0014; **f**, *P* = 0.0003; **g**, left, ns = 0.8633, ***P = 0.002; right, ns = 0.2021, ****P < 0.0001; **i**–**k**, *P* < 0.0001. Source data

**Extended Data Fig. 8:**
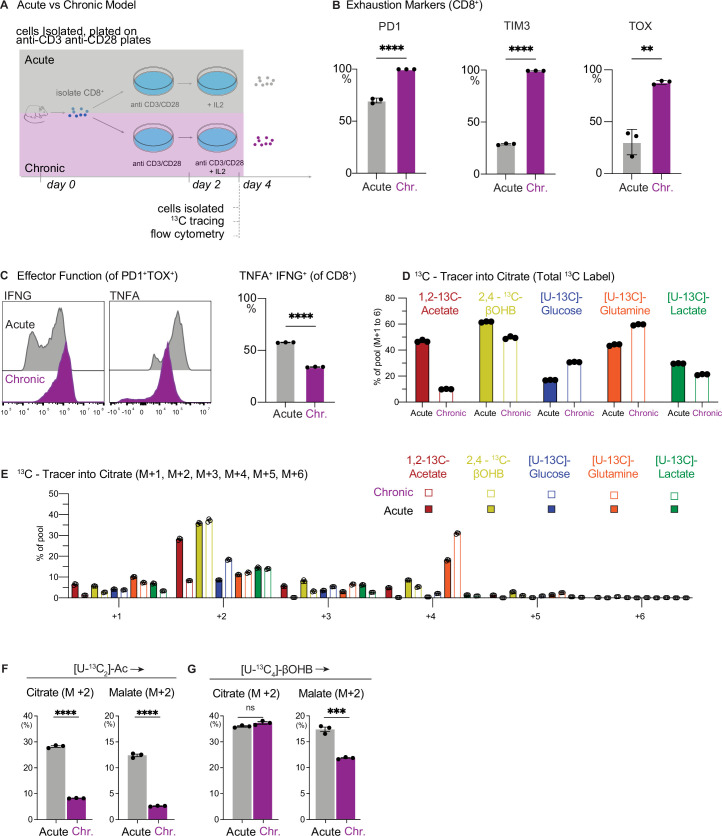
In-vitro chronic stimulation induces CD8^+^ T cell exhaustion and decreases effector function. **A**) Schematic illustrating the acute versus chronic in vitro stimulation assay, adapted from (*5*). CD8⁺ T cells isolated from C57BL/6 mice were activated with anti-CD3 and anti-CD28 antibodies (1 μg/mL and 3 μg/mL, respectively) for two days. “Acute” cells were removed from the activation plate and cultured with IL-2, while “chronic” cells were replated on fresh anti-CD3 and anti-CD28-coated plates for an additional 2–4 days. Cells were then assessed for markers of T cell exhaustion, activation, and effector function. **B**) Inhibitory receptor expression on in vitro-stimulated T cells. Bar graphs of (PD1, TIM3, TOX) percent expression level for each exhaustion marker. Data represent the mean ± SEM (n = 3 mice per group). Statistical significance was calculated via two-tailed, unpaired Student’s t-test. **C**) Representative histograms of effector cytokine production (IFN-γ and TNF-α) in CD8⁺ T cells under acute and chronic stimulation conditions. *Right*, bar plot showing the percentage of TNF⁺IFN-γ⁺ double-positive CD8⁺ T cells under acute and chronic stimulation conditions. Data represent the mean ± SEM (n = 3 mice per group). Statistical significance was calculated via two-tailed, unpaired Student’s t-test. **D**) Bar graph illustrating the fraction of ^13^C-labeled citric acid (calculated as 100% minus the unlabeled fraction, M + 0) from multiple ^13^C-labeled substrates—acetate, β-hydroxybutyrate (βOHB), glucose, glutamine, and lactate—in in vitro-stimulated CD8^+^ T cells under acute or chronic conditions. Data represent the mean ± SEM (n = 3 mice per group). **E**) Bar graph illustrating the isotopomer distribution (M + 1 through M + 6) of ^13^C-labeled citric acid derived from five different ^13^C tracers—acetate, β-hydroxybutyrate (βOHB), glucose, glutamine, and lactate—in in-vitro-stimulated CD8^+^ T cells under acute or chronic conditions. The percentage of each isotopomer is shown on the y-axis. Data represent the mean ± SEM (n = 3 mice per group). **F-G**) ^13^C labeling of TCA cycle intermediates in CD8⁺ T cells exposed to acute and chronic antigen stimulation. CD8⁺ T cells exposed to acute versus chronic stimulation as in (A) were cultured in VIM medium containing 0.4 mM [^13^C_2_]-Acetate or 0.85 mM [^13^C_4_]-βOHB for 2 h. **F**) Bar graphs of the Mass Isotopomer distribution (MID) for [^13^C_2_]-Acetate -derived citrate (M + 2) and malate (M + 2) are shown. **G**) Bar graphs of the Mass Isotopomer distribution (MID) of [^13^C_2_]-Acetate -derived citrate (M + 2) and malate (M + 2) are shown. Data represent the mean ± SEM (n = 3 mice per group). Statistical significance was calculated via two-tailed, unpaired Student’s t-test. *P < 0.05, **P < 0.01, ***P < 0.001, ****P < 0.0001. **Exact P-values are as follows:** Extended Data Fig. 8B, left, *P* < 0.0001, middle, *P* < 0.0001, right, *P* = 0.0013; Extended Data Fig. 8C, *P* < 0.0001; Extended Data Fig. 8F, left, *P* < 0.0001, right, *P* < 0.0001; Extended Data Fig. 8G, *left*, P = 0.0631, right, P = 0.0003. Source data

Chronic exposure of CD8^+^ T cells to tumour antigens in the TME is a precursor to terminal exhaustion. We therefore modelled this process using an in vitro chronic stimulation system and assessed metabolic changes on T cell self-renewal and terminal differentiation (Extended Data Fig. 8a)^20^. Chronic stimulation of in vitro-activated CD8^+^ T cells with anti-CD3 and anti-CD28 antibodies promotes several features of T cell exhaustion, including increased expression of TOX and inhibitory receptors (that is, PD1, TIM3) (Fig. 5a and Extended Data Fig. 8b), as well as reduced cytokine polyfunctionality (TNF and IFNγ production; Extended Data Fig. 8c), compared to acutely stimulated CD8^+^ T cells maintained in IL-2. Notably, chronically stimulated CD8^+^ T cells displayed increased abundance of TCA cycle intermediates compared to acutely stimulated T cells (Fig. 5b).

The increase in TCA cycle intermediates in chronically stimulated CD8^+^ T cells was similar to that observed in vivo under DR (Fig. 2h), leading us to speculate that chronic antigen stimulation alters T cell nutrient use. Using different ^13^C-labelled metabolic tracers at their normal physiologic concentrations^21^, we found that βOHB and glutamine were the greatest contributors to citrate production under chronic stimulation in vitro (Extended Data Fig. 8d), with ^13^C-βOHB contributing predominantly to the first turn of the TCA cycle (M + 2 citrate; Extended Data Fig. 8e). In vitro chronic stimulation promoted increased ^13^C-βOHB uptake by CD8^+^ T cells compared to controls (Fig. 5c and Supplementary Table 9), while ^13^C-labelled glucose uptake and entry into glycolysis (measured by formation of ^13^C-labelled glucose-6-phosphate) was unchanged between conditions (Fig. 5d). Chronically stimulated T cells displayed higher βOHB oxidation in the TCA cycle as determined by increased levels of ^13^C-βOHB-derived citrate and malate compared to controls (Fig. 5e). Although both acetate and βOHB are major fuels for citrate production in T_eff_ cells—contributing over 60% of the carbon for citrate (M + 2) production in T_eff_ cells (Extended Data Fig. 8e)—only citrate production from βOHB was maintained under chronic stimulation conditions (Extended Data Fig. 8f,g).

Ketone bodies and acetate are two major fuel sources for acetyl-CoA production in CD8^+^ T cells^22,51^, with T_ex_ cells losing the ability to process acetyl-CoA from acetate^52^. In vitro chronically stimulated CD8^+^ T cells displayed a reduced malate-to-citrate ratio (Fig. 5f and Supplementary Table 9), suggesting a net export of βOHB-derived citrate to the cytosol for acetyl-CoA production like DR-conditioned T_eff_ cells (Fig. 4j,k). Consistent with this concept, we observed increased βOHB-derived acetyl-CoA production in chronically stimulated versus acutely activated CD8^+^ T cells based on increased acetyl (M + 2) labelling of metabolites, including the nucleotide sugar UDP-GlcNAc and acetyl-methionine (Fig. 5g). Therefore, chronic stimulation promotes metabolic rewiring of oxidative metabolism in CD8^+^ T cells, with βOHB serving as a central fuel for TCA cycle metabolism and acetyl-CoA production.

One of the defining features of CD8^+^ TILs from DR tumours is the expansion of proliferating TOX^+^ T_ex_ cells with effector-like properties (that is, T_ex_^Eff^ cells; Fig. 3c,d). To define the metabolic properties of these cells, we analysed transcriptional profiles of CD8^+^ TIL subsets from human tumours. Similar to CD8^+^ TILs from mouse tumours (Fig. 3), we identified CD8^+^ T_eff_ cells and two T_ex_ TIL clusters from human tumours (Fig. 5h). The dysfunctional or exhausted-like (T_ex_) cell populations were distinguished from each other by the expression of the proliferation marker *MKI67* (T_ex_^Eff^ vs T_ex_^Term^; Fig. 5i). T_ex_^Eff^ cells from human tumours most closely associated with the T_ex_^Eff^ TIL cluster in mice (Fig. 3a). Proliferating T_ex_^Eff^ cells (human TIL) displayed the highest ketone body metabolism (ketolysis) gene signature of all human TIL subsets (Fig. 5j), driven by increased expression of βOHB dehydrogenase (*BDH1*), which encodes the first rate-limiting enzyme required for βOHB breakdown, and 3-oxoacid CoA-transferase 1 (*OXCT1*), which converts βOHB to acetoacetate (AcAc) (Fig. 5k). Notably, both human T_eff_ and T_ex_^Eff^ cells displayed significant increases *BDH1* transcript expression (Fig. 5k), which was associated with βOHB oxidation by both acutely activated and chronically stimulated CD8^+^ T cells (Extended Data Fig. 8d). Therefore, ketolysis is a metabolic signature of human TILs with effector-like properties.

### Ketone oxidation by T cells fuels the anti-tumour effects of DR

**Fig. 6:**
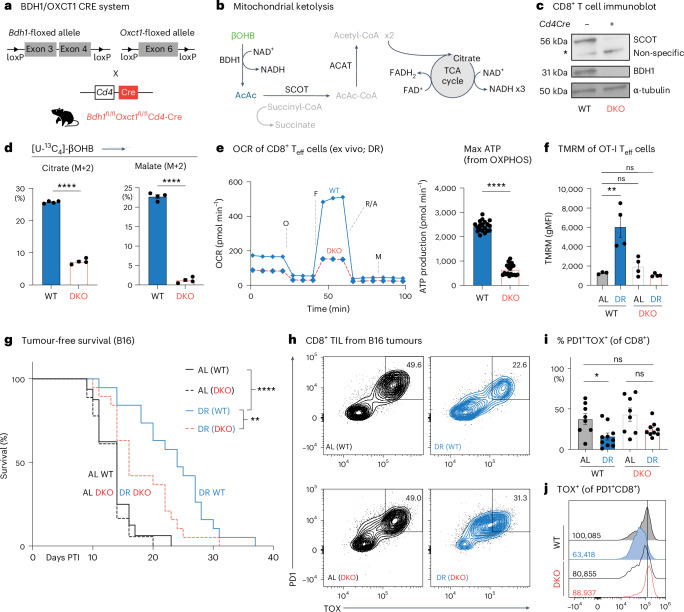
Ketone oxidation by T cells fuels the anti-tumour effects of DR. **a**, Schematic detailing the generation of *Bdh1*^fl/fl^; *Oxct1*^fl/fl^ *Cd4*-Cre mice. *Bdh1*-floxed mice were crossed with *Oxct1*-floxed mice to generate double-floxed mice expressing Cre recombinase under the *Cd4* promoter, resulting in T cells unable to metabolize ketone bodies. **b**, Diagram illustrating mitochondrial ketolysis pathways. βOHB is converted to AcAc by BDH1. AcAc is then converted to acetoacetyl-CoA by SCOT1 (*Oxct1*), ultimately leading to acetyl-CoA production for entry into the TCA cycle and ATP generation. **c**, Immunoblot of SCOT and BDH1 protein expression of CD8⁺ T cells from WT (*Cd4-*Cre^−^, *Bdh1*^fl/fl^; *Oxct1*^fl/fl^) or *Bdh1/Oxct1* DKO (*Cd4-*Cre^+^, *Bdh1*^fl/fl^; *Oxct1*^fl/fl^). Protein levels of α-tubulin are shown as a loading control. This was repeated three times independently with similar results. **d**, ^13^C labelling of TCA cycle intermediates in control (WT) and ketolysis-deficient (DKO) CD8⁺ T cells under DR conditions. Bar graph showing total labelling (pool size) U-[^13^C_4_]-βOHB labelling into citrate (left) and malate (right) in WT versus DKO CD8⁺ T cells isolated from DR-fed *Lm*OVA-infected mice (7 dpi). Data represent the means; error bars, s.e.m. (*n* = 4 mice per group). Statistical significance was calculated with a two-tailed, unpaired Student’s *t*-test. **e**, Bioenergetic profile of control (WT) and ketolysis-deficient (DKO) CD8⁺ T cells under DR conditions. Left, OCR plot of WT and DKO OT-I T cells isolated from DR-fed *Lm*OVA-infected mice (7 dpi); right, bar graph showing maximal ATP production rates from OXPHOS for WT versus DKO CD8⁺ T cells. Data represent the means; error bars, s.d. (*n* = 19 WT and 20 DKO, technical replicates). Statistical significance was calculated with a two-tailed, unpaired Student’s *t*-test. **f**, Mitochondrial membrane potential of control (WT) versus ketolysis-deficient (DKO) CD8⁺ T cells under DR conditions. Bar plot showing TMRM staining of CD8⁺ T cells isolated from *Lm*OVA-infected AL-fed or DR-fed mice (7 dpi). Data represent the means; error bars, s.e.m. (*n* = 4 mice per group). Statistical significance was calculated with two-way ANOVA with multiple comparisons. **g**, Kaplan–Meier plot comparing tumour onset (tumour volume, ≥250 mm^3^) in B16 tumour-bearing WT versus DKO mice fed an AL or DR diet as in Fig. 1b. Statistical significance was assessed by log-rank test with Bonferroni correction (*n* = 16–19 mice per group). **h**, Representative flow cytometry plots showing TOX and PD1 expression in CD8^+^ T cells from B16-OVA melanoma tumours under AL or DR conditions at 14 dpi. Four conditions are represented: AL (WT), DR (WT), AL (DKO) and DR (DKO). Data represent the means; error bars, s.e.m. (*n* = 8–10 mice per group). **i**,**j**, Enhanced exhaustion of DKO CD8⁺ T cells under DR-fed conditions. **i**, Bar plot showing the percentage of PD1⁺TOX⁺ CD8⁺ T cells isolated from B16 tumours from WT versus DKO mice under AL-fed or DR-fed conditions. **j**, Representative histograms of TOX expression in CD8⁺ TIL isolated from B16 tumours from WT and DKO mice fed under AL or DR conditions. Inset, gMFI values for TOX expression averaged across all biological replicates. Data represent the means; error bars, s.e.m. (*n* = 8 AL-WT, 8 AL-DKO, 10 DR-WT and 9 DR-DKO mice per group). Statistical significance was calculated via two-way ANOVA multiple comparisons. *P < 0.05; **P < 0.01; ***P < 0.001; ****P < 0.0001. Exact *P* values are as follows: **d**, left, *P* < 0.0001; right, *P* < 0.0001; **e**, *P* < 0.0001; **f**, top, ns = 0.9982, middle, ns = 0.5507, bottom, **P = 0.0012; **g**, ****P < 0.0001, **P = 0.0132; **i**, AL-WT vs DR-WT, *P* = 0.0221; AL-WT vs DR-DKO, *P* = 0.2737; AL-DKO vs DR-DKO, *P* = 0.0766. Source data

**Extended Data Fig. 9:**
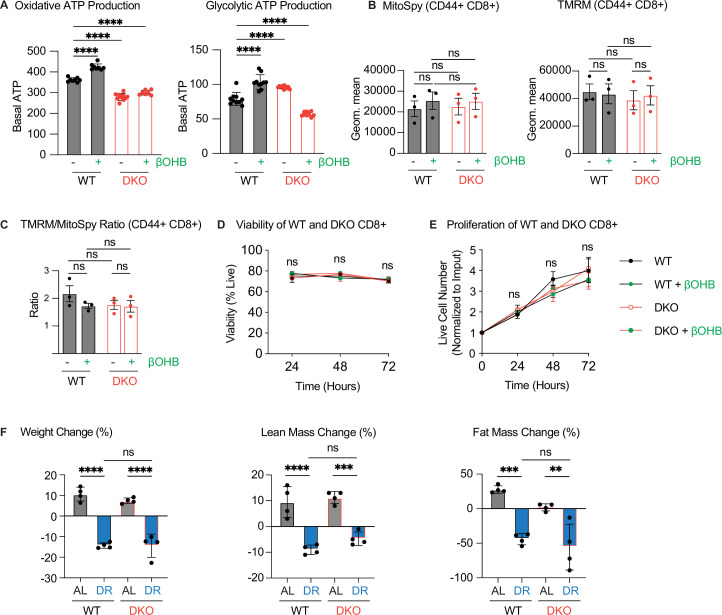
Ketolysis-deficient T cells exhibit impaired metabolic function and fail to respond to dietary restriction. **A**) Basal ATP production rates from oxidative phosphorylation (OX; derived from OCR) and glycolysis (GLY; derived from ECAR) in CD8⁺ T cells. WT (black) and DKO (red) cells were activated for 72 h on plate-bound anti-CD3/CD28 in the presence of 1.5 mM βOHB, then assayed on a Seahorse XF analyzer. Each point is one mouse (*Left*, n = 8 WT - βOHB, 8 WT + βOHB, 9 DKO - βOHB, and 8 DKO + βOHB. *Right*, n = 10 technical replicates/group), bars are mean ± SEM. Statistical significance was calculated via two-way ANOVA multiple comparisons test. **B**) Mitochondrial mass in activated CD44⁺ CD8⁺ cells, measured by MitoSpy (geometric mean fluorescence intensity, gMFI). *Right*, mitochondrial membrane potential in the same population, measured by TMRM (gMFI). Statistical significance was calculated via two-way ANOVA multiple comparisons test. **C**) Ratio of TMRM to MitoSpy gMFI, indicating membrane potential per mitochondrial unit. For panels (B–C), each bar is mean ± SEM (n = 3 mice per group); no significant differences were observed. Statistical significance was calculated via two-way ANOVA multiple comparisons test. **D**) Viability of CD8⁺ T cells at 24, 48, and 72 h post-activation, assessed by live/dead staining. Lines show mean ± SEM (n = 3). Statistical significance was calculated via two-way ANOVA. **E**) Proliferation kinetics: live cell counts at each timepoint normalized to input at 0 h. Lines represent mean ± SEM (n = 3); neither genotype nor βOHB altered viability or proliferation. Statistical significance was calculated via two-way ANOVA. **F**) Percent change in total body weight from baseline to two weeks in WT and DKO mice under ad libitum (AL) or dietary restriction (DR; 50%). Each point is one mouse (n = 4 per group); bars are mean ± SEM. *Middle*, percent change in lean mass over the same period and groups. *Right*, per cent change in fat mass over two weeks in AL vs. DR WT and DKO mice. Statistical significance was calculated via two-way ANOVA multiple comparisons test. **Exact P-values are as follows:** Extended Data Fig. 9A, **** *P* < 0.0001; Extended Data Fig. 9F:AL (WT) vs. DR (WT), *P* < 0.0001; AL (WT) vs. AL (DKO), ns = 0.567; AL (WT) vs. DR (DKO), *P* < 0.0001; DR (WT) vs. AL (DKO), *P* < 0.0001; DR (WT) vs. DR (DKO), ns > 0.9999; AL (DKO) vs. DR (DKO), *P* < 0.0001. Extended Data Fig. 9F: AL (WT) vs. DR (WT), *P* < 0.0001; AL (WT) vs. AL (DKO), ns = 0.9007; AL (WT) vs. DR (DKO), *P* = 0.0006; DR (WT) vs. AL (DKO), *P* < 0.0001; DR (WT) vs. DR (DKO), ns = 0.3824; AL (DKO) vs. DR (DKO), *P* = 0.0002. Extended Data Fig. 9F: AL (WT) vs. DR (WT), *P* = 0.0004; AL (WT) vs. AL (DKO), ns = 0.2104; AL (WT) vs. DR (DKO), *P* = 0.0001; DR (WT) vs. AL (DKO), *P* = 0.0132; DR (WT) vs. DR (DKO), ns = 0.793; AL (DKO) vs. DR (DKO), *P* = 0.0027. Source data

**Extended Data Fig. 10:**
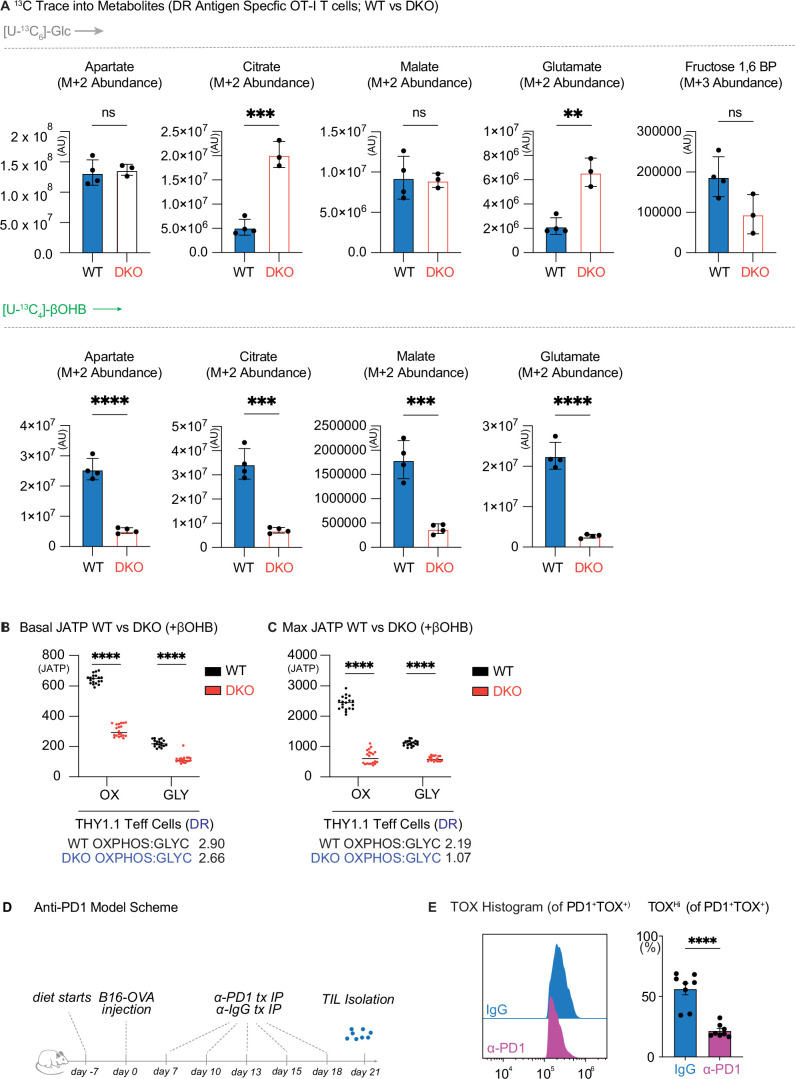
Ketolysis-deficient T cells exhibit impaired metabolic function and fail to respond to dietary restriction. **A**) DR antigen-specific OT-I CD8⁺ T cells from WT (blue) and BDH1/OXCT1 DKO (red) mice were incubated with either U-^13^C_6_-glucose (top row) or U-^13^C_4_-β-hydroxybutyrate (bottom row), and the incorporation of label into aspartate, citrate, malate, glutamate (M + 2 isotopologues) and fructose-1,6-bisphosphate (M + 3, glucose only) was quantified as isotopologue abundance × pool size. Bars show mean ± SEM (*top*, n = 3 AL and 4 DR mice per group. *Bottom*, 4 mice/group). Statistical significance was calculated via two-tailed, unpaired Student’s t-test. **B**) Basal ATP production rates (J_ATP) from oxidative phosphorylation (OXPHOS) and glycolysis (GLY) in antigen-specific CD8⁺THY1.1⁺ T cells from wild-type (WT) and double knockout (DKO; *Bdh1*^fl/fl^*Oxct1*^fl/fl^ Cd4-Cre) mice under DR conditions. T cells were isolated from LmOVA-infected mice at 7 dpi. Data represent the mean ± SEM (n = 19 WT (OXY and GLY) and 20 DKO (OXY and GLY), technical replicates). Statistical significance was calculated via two-way ANOVA multiple comparisons test. **C**) Maximal ATP production rates (J_ATP) from OXPHOS and GLY under the same conditions as in (B). Data represent the mean ± SEM (n = 19 WT (OXY and GLY) and 20 DKO-OXY and 19 DKO-GLY), technical replicates). Statistical significance was calculated via two-way ANOVA multiple comparisons test. *P < 0.05, **P < 0.01, ***P < 0.001, ****P < 0.0001. **D**) Schematic representation of anti-IgG and anti-PD1 antibody treatment in mice bearing B16 melanoma tumors under ad libitum (AL) or dietary restriction (DR) feeding regimens. Antibody treatments (200 μg/dose) were administered every three days for a total of five injections, starting on day 7 post-tumor implantation. **E**) TOX expression in PD1⁺TOX⁺ CD8⁺ TILs: left, representative flow cytometry histograms for IgG- (blue) versus α-PD-1-treated (magenta) mice; right, percentage of TOX^Hi^ cells within the PD1⁺TOX⁺ gate. Each symbol is one mouse (n = 8 per group); bars show mean ± SEM. Statistical significance was calculated via two-tailed, unpaired Student’s t-test. **Exact P-values are as follows:** Extended Data Fig. 10A, ¹³C Glucose tracing: Aspartate, *P* = 0.7503; Citrate, *P* = 0.0002; Malate, *P* = 0.85; Glutamate, *P* = 0.0014; Fructose 1,6-bisphosphate, *P* = 0.0557. ¹³C βHB tracing: Aspartate, *P* < 0.0001; Citrate, *P* = 0.0004; Malate, *P* = 0.0004; Glutamate, *P* < 0.0001. Extended Data Fig. 10B, left, *P* < 0.0001, right, *P* < 0.0001; Extended Data Fig. 10C, left, *P* < 0.0001, right, *P* < 0.0001; Extended Data Fig. 10E, *P* < 0.0001. Source data

Next, we assessed the contribution of βOHB metabolism by T cells to the anti-tumour effects of DR. To test this effect, we generated mice with T cell-specific deletion of the enzymes required to process ketone bodies—βOHB dehydrogenase 1 (BDH1) and succinyl-CoA:3-ketoacid CoA-transferase (SCOT; encoded by *Oxct1*)—by crossing *Cd4-*Cre transgenic mice to mice with floxed alleles targeting the *Bdh1* and *Oxct1* genes (*Bdh1*^fl/fl^; *Oxct1*^fl/^^fl^ *Cd4-*Cre; Fig. 6a). BDH1 mediates the conversion of βOHB to AcAc, while SCOT converts AcAc to acetoacetyl-CoA, ultimately leading to the production of acetyl-CoA that enters the TCA cycle (Fig. 6b). Depletion of BDH1 and SCOT proteins in double-knockout (DKO) T cells (from *Bdh1*^fl/fl^; *Oxct1*^fl/fl^ *Cd4-*Cre^+^ mice) was confirmed by immunoblot (Fig. 6c). Deletion of BDH1 and SCOT ablated the βOHB-induced increase in oxidative ATP production in CD8⁺ T cells (Extended Data Fig. 9a). Mitochondrial mass and mitochondrial membrane potential were similar between activated wild-type (WT) and DKO CD8⁺ T cells (Extended Data Fig. 9b,c), as were cell viability and proliferation kinetics over 72 h of activation (Extended Data Fig. 9d,e). EchoMRI analysis of control and DKO mice subjected to 50% DR for 2 weeks revealed similar changes in body weight, fat mass and lean mass, regardless of genotype (Extended Data Fig. 9f). Finally, ex vivo [^13^C_6_]-glucose and [^13^C_4_]-βOHB tracing in antigen-specific CD8^+^ T cells (OT-I) isolated from DR-fed *Lm*OVA-infected hosts confirmed that DKO T cells retain the ability to use [U-^13^C_6_]-glucose for TCA cycle metabolism but fail to channel [U-^13^C_4_]-βOHB into TCA intermediates (Fig. 6d and Extended Data Fig. 10a). These results establish that deleting *Bdh1* and *Oxct1* genes in CD8⁺ T cells specifically impairs their ability to oxidize βOHB, while maintaining whole-body metabolic responses to DR feeding.

We next asked whether ketone-body oxidation fuels the increase in CD8⁺ T cell bioenergetics induced by DR. Seahorse analysis of control (WT, from Cre^−^ *Bdh1*^fl/fl^*Oxct1*^fl/fl^ mice) or DKO (Cre^+^) OT-I CD8^+^ T cells isolated from *Lm*OVA-infected mice demonstrated that loss of BDH1 and SCOT reversed the boost in OXPHOS and ATP production induced by DR feeding (Fig. 6e and Extended Data Fig. 10b,c). TMRM staining of WT and DKO CD8^+^ T_eff_ cells revealed that the increase in mitochondrial membrane potential induced by DR was also dependent on T cell-intrinsic ketolysis (Fig. 6f). Together, these data indicate that T cell-intrinsic ketone body oxidation drives the heightened bioenergetic capacity of CD8^+^ T cells induced by DR feeding.

Finally, we assessed the contribution of T cell-intrinsic ketolysis to the anti-tumour effects of DR by challenging control (that is, WT) or DKO mice with syngeneic B16 melanoma tumours. We observed no difference in B16 melanoma tumour growth between DKO and control mice under AL conditions; however, tumour growth was significantly accelerated in DKO mice specifically under DR conditions (Fig. 6g). Analysis of CD8^+^ TILs from these mice revealed that the diet-induced change in CD8^+^ T cell fate within tumours was altered when T cells could not process ketone bodies. Tumours grown in DKO mice displayed a greater accumulation of PD1^hi^TOX^hi^ T_ex_ cells under DR when compared to WT animals on the same diet (Fig. 6h,i), with ketolysis-deficient (that is, DKO) CD8^+^ TILs maintaining high TOX levels even under DR feeding conditions that normally reduce TOX expression in WT CD8^+^ TILs (Fig. 6j). Together, these data link T cell-intrinsic ketone body oxidation to the anti-tumour effects of DR, with CD8^+^ TILs unable to process ketone bodies displaying increased features of exhaustion.

### DR synergizes with anti-PD1 blockade to enhance anti-tumour immunity

**Fig. 7:**
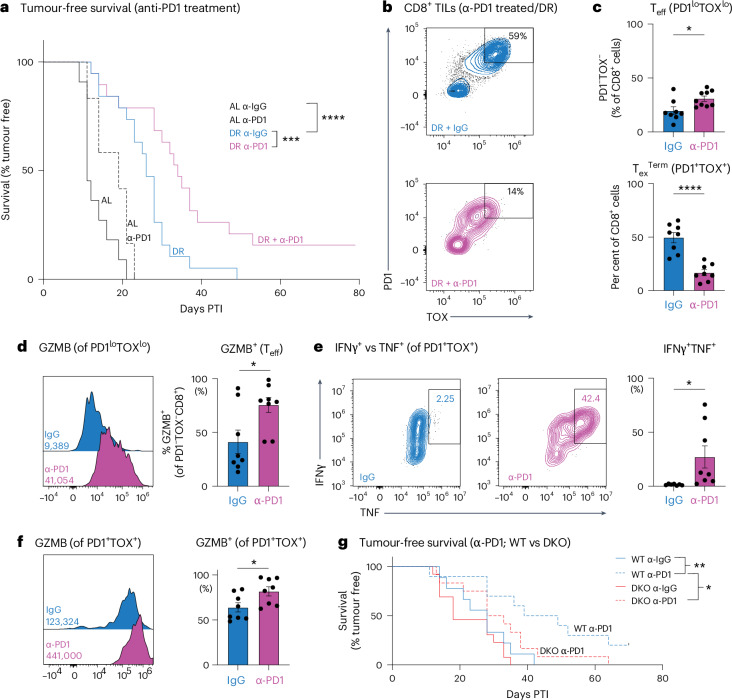
DR synergizes with anti-PD1 immunotherapy to enhance anti-tumour immunity. **a**, Kaplan–Meier plot comparing B16 melanoma tumour onset in AL-fed or DR-fed mice that received anti-PD1 or IgG control antibodies by intraperitoneal injection (200 μg per dose). Antibody treatment was administered every 3 days for a total of five injections, beginning 7 days PTI (*n* = 11–20 mice per group). Statistical significance was assessed by a log-rank test with Bonferroni correction. **b**, PD1 and TOX expression in B16 tumour-infiltrating CD8⁺ T cells from DR-fed mice treated with anti-PD1 immunotherapy. DR-fed mice harbouring B16 tumours were administered anti-IgG or anti-PD1 antibodies (200 μg intraperitoneally) every 3 days for five doses, beginning on day 7 PTI. CD8⁺ T cells were isolated from B16 melanoma tumours at 21 days PTI. Representative flow cytometry plots for TOX versus PD1 expression. **c,** Bar plot representing the per cent of T_eff_ cells (PD1^−^TOX^−^CD8^+^ TILs). Data represent the means; error bars, s.e.m. (*n* = 8 mice per group). Bottom, bar plot representing the per cent of T_ex_^Term^ cells (PD1^+^TOX^+^CD8^+^ TILs). Data represent the means; error bars, s.e.m. (*n* = 8 mice per group). Statistical significance was calculated with a two-tailed, unpaired Student’s *t*-test. **d**, Representative histograms of GZMB expression of T_eff_ from DR-fed mice harbouring B16 tumours that were administered anti-IgG or anti-PD1 antibodies. Inset, gMFI values for GZMB. Data represent the means; error bars, s.e.m. (*n* = 8). Right, bar graph showing the percentage of PD1^−^TOX^−^ CD8⁺ T cells (T_eff_) expressing GZMB following IgG or anti-PD1 treatment. Data represent the means; error bars, s.e.m. (*n* = 8 mice per group). Statistical significance was calculated with a two-tailed, unpaired Student’s *t*-test. **e**, Representative flow cytometry plots of TNF versus IFNγ expression of PD1^+^TOX^+^ CD8^+^ TILs (T_ex_^Term^) from DR-fed mice harbouring B16 tumours that were administered anti-IgG or anti-PD1 antibodies. Right, bar graphs quantifying the percentage of PD1^+^TOX^+^ CD8⁺ T cells expressing IFNγ and TNF (IFNG⁺TNF⁺). Statistical significance was calculated with a two-tailed, unpaired Student’s *t*-test. Data represent the means; error bars, s.e.m. (*n* = 7 for AL and *n* = 8 for DR). **f**, Representative histograms of GZMB expression of T_ex_^Term^ T cells from DR-fed mice harbouring B16 tumours that were administered anti-IgG or anti-PD1 antibodies from **a**. Inset, gMFI values for GZMB. Data represent the means; error bars, s.e.m. (*n* = 8). Right, bar graph showing the percentage of GZMB production from T_ex_^Term^ CD8^+^ TILs that were previously treated with anti-IgG or anti-PD1 from **a**. Data represent the means; error bars, s.e.m. (*n* = 8 mice per group). Statistical significance was calculated with a two-tailed, unpaired Student’s *t*-test. **g**, Kaplan–Meier analysis of tumour-free survival in dietary-restricted WT and ketolysis-deficient DKO mice (*Cd4-*Cre driven) treated with anti-PD1 or isotype control IgG. DR-conditioned WT (blue) and DKO (red) mice bearing subcutaneous B16 melanoma allografts received either control IgG (solid lines) or anti-PD1 antibody (dashed lines) once tumours reached 7 days PTI. Survival is plotted as days PTI. *n* = 9–13 mice per group. Statistical significance was assessed by a log-rank test. *P < 0.05; **P < 0.01; ***P < 0.001; ****P < 0.0001. Exact *P* values are as follows: **a**, AL (IgG) to DR (IgG), *P* < 0.0001; DR (IgG) to DR (anti-PD1), *P* = 0.005; **c**, top, *P* = 0.0175; bottom, *P* < 0.0001; **d**, *P* = 0.0157; **e**, *P* = 0.0376; **f**, *P* = 0.0279; **g**, WT (IgG) to WT (anti-PD1), *P* = 0.0083; WT (anti-PD1) to DKO (anti-PD1), *P* = 0.0391. Source data

Overcoming resistance to ICI therapy is a major challenge for cancer treatment. Given that DR enhances T cell-mediated control of tumour growth, we tested the efficacy of combining DR with anti-PD1 immunotherapy in mice bearing B16 melanoma tumours, which display resistance to PD1 blockade^53^. AL-fed or DR-fed C57BL/6J mice were administered anti-PD1 or IgG control antibodies beginning once tumours were palpable (Extended Data Fig. 10d). Anti-PD1 treatment promoted a small but significant increase in tumour-free survival under AL conditions; however, DR feeding greatly enhanced the efficacy of PD1 blockade, extending the tumour-free survival of animals over both anti-PD1 treatment under AL feeding and DR feeding alone (Fig. 7a). Strikingly, ~15% of animals on DR treated with anti-PD1 immunotherapy remained tumour-free after 80 days (Fig. 7a).

Proliferative T_ex_ cells expressing LY108/SLAMF6 are highly responsive to ICI therapy^4,54,55^. Given that DR synergizes with anti-PD1 treatment to slow tumour growth (Fig. 7a), we investigated the impact of DR on anti-tumour CD8^+^ T cell populations following ICI treatment. B16 tumour-bearing C57BL/6J mice subjected to DR were administered either anti-PD1 or control IgG antibodies 7 days post tumour implantation, and CD8^+^ TIL phenotypes were analysed after 15 days of treatment (as in Extended Data Fig. 10d). Combining DR with anti-PD1 treatment shifted the effector versus exhausted CD8^+^ TIL populations within the tumours (Fig. 7b). Specifically, PD1 blockade promoted an increase in CD8^+^ T_eff_ (PD1^lo^TOX^lo^) cells and an approximately threefold decrease in the frequency of CD8^+^ T_ex_ (TOX^+^PD1^+^) cells within tumours compared to DR treatment alone (Fig. 7c). PD1^lo^ TILs from anti-PD1-treated mice displayed elevated GZMB production on a per-cell basis (Fig. 7d). Conversely, TOX-expressing CD8^+^ TILs from DR-fed mice were more functional following anti-PD1 treatment, expressing lower levels of TOX (Extended Data Fig. 10e) and displaying increased cytokine polyfunctionality (that is, IFNγ^+^TNF^+^ cells; Fig. 7e) and GZMB production (Fig. 7f). Importantly, the synergy between anti-PD1 treatment and DR feeding was abolished when T cells were unable to process ketone bodies; under DR feeding, DKO mice treated with anti-PD1 antibodies showed no extension in tumour-free survival compared with IgG controls (Fig. 7g). These findings reveal that ketone body oxidation is essential for diet-induced enhancement of CD8⁺ T cell-mediated anti-tumour responses triggered by immune checkpoint blockade.

## Discussion

The tumour-suppressive effects of reduced calorie intake have long been presumed to act directly on cancer cells. Here, we demonstrate that DR works more broadly to limit tumour growth by stimulating CD8^+^ T cell-mediated anti-tumour immunity. We show that DR induces a profound reprogramming of CD8^+^ T cells in the TME, promoting the expansion of tumour-controlling effector (T_eff_-like and T_ex_^Eff^) cells while limiting terminal T cell exhaustion. DR enhances CD8^+^ T cell-mediated anti-tumour immunity by increasing circulating ketone body levels, which in turn enhance TCA cycle metabolism and mitochondrial bioenergetics of CD8^+^ T cells. T cells that cannot metabolize ketone bodies display metabolic deficits, undergo premature exhaustion and fail to control tumour growth under DR conditions, identifying T cell-intrinsic ketolysis as a central mechanism for the anti-tumour effects of DR. These findings, along with our previous research identifying ketolysis as a regulator of CD8^+^ T cell cytolytic function^22^, suggest that DR regulates a nutrient-sensitive checkpoint within the TME—mediated by ketone bodies—that promotes the expansion of tumour-controlling effector (T_eff_-like and T_ex_^Eff^) T cells over terminal exhaustion, thereby improving tumour control. Overall, our study highlights how altering systemic nutrient availability through diet can influence CD8^+^ T cell fate within tumours to limit cancer progression.

βOHB is preferentially oxidized over glucose for ATP production in CD8^+^ T cells and boosts T cell effector responses^22,49^. Our data indicate that ketolysis is a metabolic signature of T_ex_ cells and that βOHB uptake and oxidation is prioritized by chronically stimulated CD8^+^ T cells to fuel bioenergetic and biosynthetic processes. Increased *BDH1* expression in CD8^+^ T cells, which we observed in TILs from human tumours, facilitates greater βOHB oxidation in TILs as they infiltrate solid tumours. Many tumours are nutrient-poor^24,56^. By increasing ketone body availability approximately fourfold compared to AL*-*fed conditions, DR boosts the available βOHB supply in the TME, fuelling the TCA cycle to meet the oxidative demands of CD8^+^ TILs. Consistent with this observation, CD8^+^ T cells that are adapted to DR display increased mitochondrial membrane potential and oxidative ATP production. This metabolic advantage promotes the expansion of T_ex_^Eff^ cells, which retain proliferative capacity and effector function, over T_ex_^Term^ cells, which have high inhibitory receptor expression and diminished functionality. These metabolic advantages are lost in ketolysis-deficient (that is, DKO) T cells, contributing to their premature exhaustion. Thus, DR aligns systemic nutrient supply with the metabolic needs of T cells at the tissue level.

Acetyl-CoA is a critical metabolite for CD8^+^ T cell effector function, and failure to produce sufficient levels of cytosolic acetyl-CoA is a precursor for terminal exhaustion^52^. Additionally, diet directly impacts acetyl-CoA homoeostasis in CD8^+^ T cells, as acetyl-CoA levels in T cells double under DR-fed conditions. Our data indicate that chronic T cell receptor stimulation enhances acetyl-CoA production through βOHB oxidation and suggest that DR may enhance acetyl-CoA production through this route by increasing circulating ketone body levels. Acetyl-CoA has functions in addition to biosynthetic growth (that is, de novo lipogenesis) and energy production: it is the limiting substrate for histone acetylation reactions that regulate T cell differentiation and effector function^51,57^. Our results argue that nutritional regulation of ketone body metabolism is a critical determinant of CD8^+^ T cell fate in the TME, shifting differentiation between terminally exhausted (T_ex_^Term^) and effector-like (T_ex_^Eff^) states. Consistent with this idea, pantothenate/CoA increases CD8^+^ T cell differentiation towards effector lineages to enhance tumour control^23^. We speculate that dietary interventions or therapeutics that boost acetyl-CoA production in T cells, such as DR, limit tumour growth by driving CD8^+^ T cell differentiation towards effector lineages and away from dysfunctional states.

The mammalian immune system evolved under cyclical periods of feast and famine. Immune challenges such as infection and cancer further impact systemic nutrient availability by disrupting feeding behaviours and remodelling host metabolism^58–60^. Mobilizing stored energy from adipose tissue into ketone body production provides the host with a versatile fuel to maintain acetyl-CoA production under conditions of metabolic stress^61,62^. In this vein, we speculate that T cells evolved the use of ketolysis to buffer against metabolic perturbations that negatively impact T cell bioenergetics and function. Our findings reveal the potential to exploit this system through nutritional interventions that enhance anti-tumour immunity. PD1 blockade amplifies the anti-tumour effects of DR by promoting the expansion of effector T cells (T_eff_ and T_ex_^Eff^ cells) to limit tumour growth, highlighting the potential to enhance the efficacy of immunotherapies through nutritional intervention.

Using DR in clinical settings may face challenges regarding feasibility owing to patient health and compliance. However, combining ICIs with pharmacological agents that reduce appetite and food intake, such as GLP-1 agonists like semaglutide (that is, Ozempic), may mimic some metabolic effects of DR without necessitating strict dietary regimens^63^. Our findings also have potential implications for adoptive T cell therapies, such as chimeric antigen receptor T cell therapy, in which the metabolic state of T cells before infusion is critical for their persistence, functionality and anti-tumour efficacy in patients^64^. Exposing chimeric antigen receptor T cells to a DR-like environment during expansion or enhancing their capacity to oxidize ketone bodies may improve their metabolic fitness and resistance to terminal exhaustion in vivo, thereby increasing therapeutic efficacy. Understanding how dietary interventions such as DR impact T cell metabolism and differentiation fate in the TME may help inform evidence-based nutritional guidelines that complement or enhance current cancer immunotherapy strategies.

### Limitations of the study

Our findings establish a role for DR in reprogramming CD8⁺ T cell fate and function in transplantable syngeneic tumour models (that is, B16 melanoma, EO771 breast cancer). Although these models are useful for mechanistic evaluation of anti-tumour T cell responses, they do not fully capture tumour evolution, stromal remodelling or the cellular and antigenic heterogeneity characteristic of human tumours. The effects of DR on anti-tumour T cell function in spontaneously arising or genetically engineered mouse tumour models, therefore, remain to be determined. Although we demonstrate that CD8⁺ T cells are required for the anti-tumour effects of DR, the *Cd4*-Cre model used in our study deletes *Bdh1* and *Oxct1* in both CD4⁺ and CD8⁺ T cell lineages; therefore, we cannot exclude a contribution of ketolysis in CD4⁺ T cells to the observed effects. In addition, metabolic analysis of CD8⁺ TILs was limited by the number of cells that could be recovered from tumours. To overcome this limitation, we used our established *Lm*OVA infection model to examine the metabolism of DR-conditioned T_eff_ cells ex vivo; however, this system may not fully capture the nutritional and environmental cues of the TME under DR. We also show that DR elevates circulating and intratumoral βOHB, but the relative impact of DR compared to other methods of increasing ketones (for example, exogenous ketone esters or ketogenic diets) on CD8⁺ T cell anti-tumour function remains unclear. Finally, although effector-like T_ex_ cells isolated from human tumours display a prominent ketolysis signature, the available datasets lack donor-matched dietary and metabolic metadata (for example, fasting state, BMI, βOHB levels). Consequently, these data cannot be used to establish a causal relationship between diet and TIL phenotype in patients.

## Methods

### Ethical compliance

All research described in this study was conducted in accordance with relevant institutional and national ethical guidelines and regulations. All animal experiments were approved by the Institutional Animal Care and Use Committee of the Van Andel Institute (VAI) and performed in compliance with institutional and federal regulations governing the humane treatment of research animals.

### Experimental model and subject details

#### Mice

This study used the following mouse strains: C57BL/6J (RRID: IMSR_JAX:000664); B6.PL-*Thy1*^*a*^/CyJ (Thy1.1; RRID: IMSR_JAX:000406); B6.SJL-*Ptprc*^*a*^ *Pepc*^*b*^/BoyJ (CD45.1^+^); and Tg(TcraTcrb)1100Mjb (OT-I; RRID: IMSR_JAX:003831), all ordered from The Jackson Laboratory. The *Bdh1*^fl/fl^; *Oxct1*^fl/fl^*Cd4*-Cre line was generated by crossing *Bdh1*^fl/fl^*Cd4*-Cre mice^22^ and *Oxct1*-floxed mice (provided by P. Crawford^65,66^). *Bdh1*^fl/fl^; *Oxct1*^fl/fl^ *Cd4*-Cre OT-I mice were generated by crossing *Bdh1*^fl/fl^; *Oxct1*^fl/fl^*Cd4*-Cre mice with the Tg(TcraTcrb)1100Mjb mouse line. All mice were bred and housed under specific-pathogen-free conditions at VAI, following approved protocols. Genotyping was conducted using DNA extracted from tail or ear biopsies, with primer sets listed in the Key Resources Table. The study included both male and female mice aged 8–14 weeks.

#### Cell lines

B16-F10 murine melanoma cells expressing OVA (B16-OVA^67^) and EO771 breast cancer cells (CRL-3461) were cultured in DMEM from Wisent, supplemented with 10% heat-inactivated FBS, 1% penicillin–streptomycin (Gibco) and 6 mM L-glutamine. All cell cultures were maintained in a humidified incubator at 37 °C with 5% CO_2_.

#### Tumour models

Male and female C57BL/6J, *Bdh1*^fl/fl^; *Oxct1*^fl/fl^ (WT), or *Bdh1*^fl/fl^; *Oxct1*^fl/fl^ *Cd4*-Cre (DKO) mice were maintained on a 5010 diet. Mice fed AL were allowed free access to food at all times and never ran out of food. The mice on the DR regimen received 50% of their average daily (normal) intake of food. Mice on DR were given pre-weighed pellets between 09:00 h and 11:00 h every day of the experiment. Following 1 week on their respective diet, mice were subcutaneously injected with 2.5 × 10^5^ (EO771) or 5 × 10^5^ (B16-OVA) cells into the right abdominal flank. Tumour volume was measured every 2–3 days with a calliper once tumours became palpable. Tumour initiation was scored as a tumour volume of ≥250 mm^3^. Mice were euthanized when the tumour volume reached 1,500 mm^3^. For experiments involving anti-PD1 treatment, mice received 200 µg of IgG (BP0091; RRID: AB_1107773) or anti-PD1 (BP0033-2; RRID:AB_1107747) antibodies intraperitoneally every 3 days, for a total of five injections (1 mg of antibody total). Treatment was initiated 7 days after tumour cell injection. The maximal tumour size permitted by the ethical regulations was 1,500 mm^3^, and this limit was not exceeded in any experiment. Animals were monitored regularly for signs of discomfort or distress, and any animal reaching predefined humane endpoints was euthanized in accordance with approved protocols.

#### TIL isolation

TIL were isolated from palpable tumours 12–14 days post tumour cell injection. Tumours were mechanically homogenized in a six-well plate and then passed through a 100 µm cell strainer, followed by a 40 µm cell strainer. The single-cell suspension was incubated with red blood cell lysis buffer for 1 min at 20–22 °C (room temperature), after which three volumes of T cell media (TCM) were added to halt the lysis reaction. Cells were collected by centrifugation at 500 RCF for 5 min at 4 °C and then resuspended in 0.5–1 ml of TCM and processed for flow cytometry.

#### Mouse T cell isolation and culture

CD8^+^ T cells were purified from mouse spleens through negative selection using magnetic bead-based isolation kits (StemCell Technologies). T cells were cultured in TCM: Iscove’s Modified Dulbecco’s Medium (IMDM; Wisent) containing 10% Nu-Serum IV culture supplement (Corning), 50 U ml^−1^ penicillin, 50 µg ml^−1^ streptomycin (Gibco), 50 µM 2-mercaptoethanol (Gibco) and 2 mM L-glutamine (Gibco; final concentration 6 mM). T cells (1 × 10^6^ cells per ml) were activated in vitro by stimulation with plate-bound anti-CD3ε (clone 145-2C11; 2 µg ml^−1^) and anti-CD28 (clone 37.51; 1 µg ml^−1^) antibodies for 48 72 h. After activation, cells were cultured in IMDM with 50 U ml^−1^ recombinant murine IL-2 (Peprotech) and re-seeded at 4 × 10^5^ cells per ml in fresh medium supplemented with IL-2 every 2 days.

For induction of in vitro chronically stimulated cells, CD8^+^ T cells were activated in vitro for 48 h as described above, followed by culture on fresh plate-bound anti-CD3ε and anti-CD28 antibodies for an additional 48 h as previously described^20^. Acutely activated cells were maintained in IL-2 (50 U ml^−1^) without restimulation.

#### Adoptive transfer and *Lm*OVA infection

Mice were intravenously injected with a sublethal dose of recombinant *L. monocytogenes* expressing OVA (*Lm*OVA, 2 × 10^6^ CFU), following a previously established protocol^22^. For adoptive transfer studies involving analysis at 6–7 dpi, 5 × 10^3^ OT-I CD8⁺ T cells (Thy1.1⁺ or CD45.2⁺) were transferred intravenously into C57BL/6J recipients (Thy1.2⁺CD45.2⁺ or CD45.1⁺) on day −1, and *Lm*OVA infection was induced 24 h later (day 0). Splenocytes were collected at 7 dpi to evaluate OVA-specific CD8⁺ T cells through Thy1.1 or CD45.2 staining. Cytokine production was measured using intracellular cytokine staining following re-stimulation with OVA peptide (OVA_257–264_) for 4 h (GolgiStop added at 1 µg ml^−1^ after 2 h of restimulation) at 37 °C.

For metabolic analysis of *Lm*OVA-specific Thy1.1^+^ OT-I T cells ex vivo, Thy1.2^+^ C57BL/6 mice received 5 × 10^4^ Thy1.1^+^ OT-I T cells at day −1, followed by *Lm*OVA infection on day 0. *Lm*OVA-specific CD8^+^ OT-I T cells were isolated from the spleen of infected mice by positive selection using the EasySep mouse CD90.1 positive selection kit (StemCell Technologies) as previously described^43,68^. For ex vivo ^13^C isotope tracing, 5 × 10^4^ Thy1.1⁺ OT-I CD8⁺ T cells were injected into Thy1.2⁺CD45.2⁺ C57BL/6J mice, followed by *Lm*OVA infection the next day. At 7 dpi, activated Thy1.1⁺ OT-I T cells were isolated from the spleens using magnetic bead purification^43,68^ and cultured in vitro for 2 h in VIM medium^21^ containing 1.5 mM βOHB.

#### Ketone body measurements

To monitor blood ketone levels, blood was collected from the submental vein of restrained mice, and ketone test strips (Keto-Mojo) were used to quantify blood ketone levels.

#### Metabolite and lipid extraction

For T cell tracing and metabolite profiling studies, metabolites were extracted by mixing with ice-cold acetonitrile:methanol:water (4:4:2, v/v)^69^, sonicating for 5 min and incubating on wet ice for 1 h. Extracts were centrifuged, and the soluble fraction was collected and dried under vacuum. Extracts were resuspended in 50 μl of water for liquid chromatography–mass spectrometry (LC–MS) analysis. For tissue and plasma metabolite and lipid profiling, samples were extracted with chloroform:methanol:water (2:2:1.8 v/v)^69,70^. For plasma, this was accomplished by mixing 40 μl of plasma with 690 μl 1:1 chloroform:methanol. For tissue, 690 μl of 1:1 chloroform:methanol was added to 40 mg of tissue and homogenized with a beadmill homogenizer. For both sample types, 310 μl of water was added, incubated on wet ice for 1 h and centrifuged at 14,000*g* for 10 min to induce phase separation. Then, 495 μl and 100 μl of the upper aqueous and bottom organic layers, respectively, were collected into separate tubes and dried in a rotary vacuum evaporator. The aqueous layer was resuspended in 100 μl of water for metabolomics analysis. The organic layer was resuspended in 200 μl of 50:50 (isopropanol:acetonitrile, v/v) for lipidomics analysis.

#### Metabolomics analyses

Metabolomic profiling and stable isotope tracing data were collected using a Vanquish liquid chromatography system coupled to an Orbitrap Exploris 240 (Thermo Fisher Scientific) using a heated electrospray ionization (H-ESI) source in negative mode as previously described^22,69^. A total of 2 μl of each standard and/or sample was injected and run through a 24-min reversed-phase chromatography Zorbax RRHD extend-C18 column (1.8 μm, 2.1 mm × 150 mm; Agilent, 759700-902) combined with a Zorbax extend-C18 guard column (1.8 μm, 2.1 mm × 5 mm; Agilent, 821725-907). Mobile phase A consisted of LC–MS-grade water (W6, Fisher) with 3% LC–MS-grade methanol (A456, Fisher), mobile phase B was LC–MS-grade methanol and both mobile phases contained 10 mM tributylamine (Sigma-Aldrich, 90780), 15 mM LC–MS-grade acetic acid (Fisher, A11350) and 0.01% medronic acid (v/v; Agilent, 5191-4506). For the wash gradient, mobile phase A was kept the same, and mobile phase B was 99% LC–MS-grade acetonitrile (Fisher, A955). Column temperature was kept at 35 °C, flow rate was held at 0.25 ml min^−1^ and the chromatography gradient was as follows: 0–2.5 min held at 0% B; 2.5–7.5 min from 0% B to 20% B; 7.5–13 min from 20% B to 45% B; 13–20 min from 45% B to 99% B; and 20–24 min held at 99% B. A 16-min wash gradient was run in reverse flow direction between every injection to back-flush the column and to re-equilibrate solvent conditions as follows: 0–3 min held at 100% B and 0.25 ml min^−1^; 3–3.5 min held at 100% B and ramp to 0.8 ml min^−1^; 3.5–7.35 min held at 100% B and 0.8 ml min^−1^; 7.35–7.5 min held at 100% B and ramp to 0.6 ml min^−1^; 7.5–8.25 min from 100% B to 0% B and ramp to 0.4 ml min^−1^; 8.25–15.5 min held at 0% B and ramp to 0.25 ml min^−1^; and 15.5–16 min held at 0% B and 0.25 ml min^−1^. MS parameters were as follows: source voltage −2,500V; sheath gas, 60; aux gas, 19; sweep gas, 1; ion transfer tube temperature, 320 °C; and vaporizer temperature, 250 °C. Full scan data were collected using an Orbitrap with a scan range of 70–850 *m*/*z* at a resolution of 240,000 and RF lens at 35%. Fragmentation was induced in the Orbitrap using assisted higher-energy collisional dissociation (HCD) collision energies at 15, 30 and 45%. Orbitrap resolution was 15,000, the isolation window was 2 *m*/*z* and data-dependent scans were capped at five scans.

For acetyl-CoA measurements, samples were analysed with a Vanquish liquid chromatography system coupled to an Orbitrap ID-X (Thermo Fisher Scientific) using an H-ESI source in positive mode. A total of 2 μl of each standard and/or sample was injected and run through a 5-min reversed-phase chromatography Cortecs T3 Column (1.6 μm, 2.1 mm × 150 mm; Waters, 186008500) combined with a Cortecs T3 VanGuard Pre-column (1.6 μm, 2.1 mm × 5 mm; Waters, 186008508). Mobile phase A consisted of 100% LC–MS-grade water (Fisher, W6), 0.01% ammonium hydroxide (Fisher, A470) and 5 mM ammonium acetate (Sigma-Aldrich, 73594), and mobile phase B consisted of 99% LC–MS-grade acetonitrile (Fisher, A955). Column temperature was kept at 30 °C, flow rate was held at 0.3 ml min^−1^, and the chromatography gradient was as follows: 0–0.5 min held at 0% B; 0.5–1 min from 0% B to 10% B; 1–4 min from 10% B to 50% B; 4–4.1 min from 50% B to 99% B; and 4.1–5 min held at 99% B. A 5-min wash gradient was run between every injection to flush the column and to re-equilibrate solvent conditions as follows: 0–2 min held at 100% B; 2–3 min from 100% B to 0% B; and 3–5 min held at 0% B. MS parameters were: source voltage, 3,500 V; sheath gas, 70; aux gas, 25; sweep gas, 1; ion transfer tube temperature, 300 °C; and vaporizer temperature, 250 °C. A targeted single ion scan was performed in the Orbitrap to target acetyl-CoA and all of its carbon-13 isotopologues with a centre mass of 810.133 *m*/*z* and an isolation window of 48 *m*/*z*. Resolution was set at 60,000, RF lens at 60% and scan time was set for 2–4 min of the chromatographic gradient described above. Data-dependent MS2 fragmentation was induced in the Orbitrap using assisted HCD collision energies at 20, 40, 60, 80 and 100% as well as with collision-induced dissociation (CID) at a collision energy of 35%. For both MS2 fragmentations, Orbitrap resolution was 30,000, the isolation window was 1 *m*/*z* for HCD and 1.5 *m*/*z* for CID, and the total cycle time was 0.6 s.

For βOHB quantification in serum and tumours, metabolites were extracted with a 40% acetonitrile, 40% methanol and 20% water solution. An external standard curve for βOHB was generated from 10 µg ml^−1^ to 0.01 µg ml^−1^ by half-log serial dilutions. Standards were processed identically to tissue and serum samples throughout the workflow. Tissues were extracted at a concentration of 40 mg tissue per ml, and serum at 40 µl serum per ml of extraction solvent. Extracts were dried and reconstituted in water (200 µl for tissue, 1 ml for serum) containing 100 ng ml^−1^ of internal standard ([U-^13^C_4_]-βOHB). Samples and standards were analysed on an Orbitrap Exploris 240 (Thermo) in ESI-negative mode using tributylamine ion-paired chromatography as described above. Data were processed using Skyline, and internal standard-normalized peak areas were used to calculate βOHB concentrations, which are reported in units of mg g^−1^ for tissues and mM for serum.

#### Lipidomics analyses

Lipidomics samples were analysed with a Thermo Vanquish dual liquid chromatography system using two alternating methods, referred to as chromatography 1 and chromatography 2, coupled to an Orbitrap ID-X (Thermo Fisher Scientific) using an H-ESI source in positive and negative mode, respectively. A total of 2 μl of each standard and/or sample was injected, column temperatures were kept at 50 °C and the flow rate was held at 0.4 ml min^−1^. For both chromatography 1 and 2, mobile phase A consisted of 60% LC–MS-grade acetonitrile (Fisher Scientific, A955), 40% LC–MS grade water (Fisher Scientific, W6), 0.1% LC–MS-grade formic acid (Fisher Scientific, A117) and 10 mM ammonium formate (Fisher Scientific, 70221), and mobile phase B consisted of 90% LC–MS-grade isopropanol (Fisher Scientific, A461), 8% LC–MS-grade acetonitrile, 2% LC–MS-grade water, 0.1% LC–MS-grade formic acid and 10 mM ammonium formate. Chromatography 1 used a 30-min reversed-phase chromatography Accucore C30 column (2.6 μm, 2.1 mm × 150 mm; Thermo Fisher Scientific, 27826-152130) combined with an Accucore C30 guard column (2.6 μm, 2.1 mm × 10 mm; Thermo Fisher Scientific, 27826-012105), and the gradient was as follows: 0–1 min held at 25% B; 1–3 min from 25% B to 40% B; 3–19 min from 40% B to 75% B; 19–20.5 min 75% B to 90% B; 20.5–28 min from 90% B to 95% B; 28–28.1 min from 95% B to 100% B; and 28.1–30 min held at 100% B. A 30 min wash gradient was run between every injection (in parallel with chromatography 2) to flush the column and to re-equilibrate solvent conditions as follows: 0–2 min held at 100% B and 0.3 ml min^−1^; 2–2.1 min from 100% B to 25% B and held at 0.3 ml min^−1^; 2.1–4 min held at 25% B and ramp to 0.4 ml min^−1^; 4–6 min held at 25% B and ramp to 0.6 ml min^−1^; 6–17 min held at 25% B and 0.6 ml min^−1^; 17–17.1 min held at 25% B and ramp to 0.4 ml min^−1^; and 17.1–30 min held at 25% B and 0.4 ml min^−1^. Chromatography 2 used a 30-min reversed-phase chromatography Acquity UPLC CSH C18 column (1.7 μm, 2.1 mm × 100 mm; Waters, 186005297) combined with a a VanGuard pre-column (1.7 μm, 2.1 mm × 5 mm; Waters, 186005303), and the gradient was as follows: 0–1 min held at 25% B; 1–3 min from 25% B to 40% B; 3–4 min from 40% B to 50% B; 4–16 min from 50% B to 65% B; 16–17 min from 65% B to 70% B; 17–25 min from 70% B to 75% B; 25–27 min from 75% B to 100% B; and 27–30 min held at 100% B. A 30-min wash gradient was run between every injection (in parallel with chromatography 1) that used the same gradient as the chromatography 1 wash gradient. For both methods, MS parameters were as follows: source voltage, +3,250 V or −3,000 V depending on method polarity; sheath gas, 40; aux gas, 10; sweep gas, 1; ion transfer tube temperature, 300 °C; and vaporizer temperature, 275 °C. Full scan data were collected using the Orbitrap with a scan range of 200-1,700 *m*/*z*at a resolution of 500,000 and RF lens at 45%. Data-dependent MS2 fragmentation was induced in the Orbitrap using assisted HCD collision energies at 15, 30, 45, 75 and 110% as well as with CID at a collision energy of 35%. For both MS2 fragmentations, Orbitrap resolution was 15,000, and the isolation window was 1.5 *m*/*z*. A *m*/*z* 184 mass trigger, indicative of phosphatidylcholines, was used for CID fragmentation. Data-dependent MS3 fragmentation was induced in the ion trap with the scan rate set at Rapid, using CID at a collision energy of 35%. MS3 scans were triggered by specific acyl chain losses for detailed analysis of mono-, di- and triacylglycerides. Total cycle time was 2 s. Lipid identifications were assigned using LipidSearch (v.5.0; Thermo Fisher Scientific).

For data analysis, peak picking and integration was conducted in Skyline (v.22-23) using in-house curated compound lists of accurate mass MS1 and retention time of chemical standards^69^. For lipidomics studies, the Lipidsearch identifications were used to populate the target compound list for Skyline. For tracing studies, this list was expanded to include all possible carbon isotopologues, and natural abundance correction was completed using IsoCorrectR^71^.

#### In vitro acute versus chronic stimulation of T cells

Chronic stimulation of CD8^+^ T cells was conducted as previously described^20^. In brief, female P14 Thy1.1 C57BL/6 mice aged 8–12 weeks were used as a source for primary T cells. CD8^+^ (P14) cells were isolated from spleens and peripheral lymph nodes using the EasySep Mouse CD8^+^ T cell isolation kit following the manufacturer’s instructions. Following isolation and flow cytometry-based purity assessment, 5 × 10^6^ live P14 cells resuspended in TCM containing 10 ng ml^−1^ IL-2 were placed in overtop chambers of a six-well dish that were coated 24 h prior (at 4 °C) with 3 μg ml^−1^ anti-mouse CD3 (eBioscience, Clone 145-2C11) and 1 μg ml^−1^ anti-mouse CD28 (eBioscience, Clone 37.51) in 1× PBS (Wisent, 311-010-CL). After 48 h incubation (37 °C incubator, 5% CO_2_), cells were gently displaced from the coated wells, counted and 2 × 10^6^ cells were resuspended in fresh media containing 10 ng ml^−1^ IL-2 in a final volume of 5 ml in VIM medium^21^. For continuous stimulation conditions, cells were placed in three wells coated with anti-CD3 and anti-CD28 antibodies in the same fashion as mentioned above, while for acute stimulation conditions, cells were placed directly in three uncoated wells of a six-well dish in VIM containing IL-2. Then, 48 h post incubation, the media were changed, and cells were seeded in the same fashion as the previous passage.

For flow cytometric assessment of CD8^+^ T cells in vitro, 3 × 10^5^ live cells were centrifuged at 500*g* for 5 min, followed by resuspension in 200 µl TCM with 50 U ml^−1^ IL-2. Acute and continuously stimulated cells were seeded in 96-well round-bottom plates and re-stimulated with PMA (50 ng ml^−1^) and ionomycin (500 ng ml^−1^) for 4 h (37 °C incubator, 5% CO_2_), with protein transport inhibitor GolgiStop (1:1,500 dilution; BD Biosciences, 5102092KZ) added for the last 2 h, followed by processing for downstream FACS analysis. For unstimulated controls, T cells were only treated with GolgiStop for 2 h.

For ^13^C-tracing experiments, 1–2 × 10^6^ T cells were either cultured for 4 h at 37 °C in VIM medium containing IL-2 and either 5 mM ^13^C-glucose/0.85 mM ^12^C-βOHB or 5 mM ^12^C-glucose/0.85 mM ^13^C-βOHB. For unlabelled controls, T cells were cultured in VIM containing 5 mM ^12^C-glucose/0.85 mM ^12^C-βOHB. Following incubation, cells were pipetted into 15 ml conical tubes and centrifuged at 600*g* for 3 min. The medium was aspirated, then cells were washed twice with 5 ml ice-cold 0.9% w/v NaCl (saline) and spun at 600*g* for 2 min. Samples were immediately frozen on dry ice for 5 min, then transferred to a −80 °C freezer until processing for metabolomics.

#### Ex vivo stable isotope labelling and metabolomics

Ex vivo stable isotope labelling experiments with CD8^+^ T cells isolated from mice using LC or gas chromatography coupled to MS were performed as outlined previously^21,22^. Antigen-specific T cells were isolated from AL or DR *Lm*OVA-infected mice (*BDH1*/*OXCT1* DKO or WT mice) using magnetic bead isolation as previously described^22,43^. CD8^+^ T cells were cultured for 2 h in VIM medium containing 1.5 mM ^13^C_4_-βOHB, followed by metabolite extraction as described above. For ^13^C co-tracing experiments, CD8^+^ T cells were cultured for 2 h in VIM medium containing 50 U ml^−1^ IL-2 and 5 mM ^13^C_6_-glucose and 1.5 mM 2,4-^13^C_2_-βOHB (Cambridge Isotope Laboratories). Identical unlabelled controls were performed using VIM medium containing ^12^C-glucose and ^12^C-βOHB. After the 2 h incubation period, cells were centrifuged at 600*g* for 4 min, media were collected and flash-frozen over dry ice and cell pellets were washed twice with 0.9% NaCl before flash-freezing and storage at −80 °C for downstream metabolomics analysis.

#### Immunoblotting

Immunoblotting was performed by lysing cells in RIPA buffer containing protease and phosphatase inhibitors (Roche) on ice for a minimum of 30 min as previously described^72^. Protein quantification was done using the Pierce BCA Protein Assay Kit (Thermo Fisher Scientific). Equal amounts of protein from whole cell lysates were diluted in Laemmli sample buffer, boiled for 5 min, resolved on a 10% SDS–PAGE gel and transferred to nitrocellulose membranes. Membranes were blocked for 1 h at room temperature in 5% non-fat milk prepared in TBS-T, followed by overnight incubation at 4 °C with primary antibodies against BDH1 or SCOT (1:1,000 dilution in 5% non-fat milk). After washing three times in 1× TBS-T (5 min per wash), membranes were incubated for 1 h at room temperature with horseradish peroxidase-conjugated secondary antibodies (diluted in 5% non-fat milk). Membranes were washed again three times with 1× TBS-T before being developed using ECL solution (Cytiva). Details of the antibodies used are provided in the Key Resources Table.

#### Bioenergetics (Seahorse) extracellular flux assay

T cell oxygen consumption rate and extracellular acidification rate were measured using a Seahorse XF96 Extracellular Flux Analyzer, as previously described^22^. Antigen-specific Thy1.1^+^ OT-1 CD8^+^ T cells isolated from *Lm*OVA-infected mice were seeded at 2 × 10^5^ cells per well in Seahorse XF medium containing 5 mM glucose and 0.5 mM glutamine, with or without 1.5 mM βOHB (depending on experimental conditions). Cells were centrifuged onto a poly-D-lysine-coated XF96 plate, and cellular bioenergetics were evaluated at 5 min intervals after the sequential addition of oligomycin (2 µM), FCCP (1.5 µM), rotenone/antimycin A (0.5 µM each) and monensin (20 µM). Data were normalized to cell number. Bioenergetics data interpretation followed previously established protocols^73^.

#### Flow cytometry

Single-cell suspensions were prepared from mouse spleens by mechanical dissociation. Red blood cells were lysed using red blood cell lysis buffer containing 0.15 M NH₄Cl, 10 mM KHCO₃ and 0.1 mM EDTA, followed by neutralization with three volumes of TCM. For cell staining, single-cell suspensions were incubated with a cocktail of fluorescently labelled antibodies and dyes as listed in the Key Resources Table. Cell viability was assessed using Fixable Viability Dye eFluor 506 (Thermo Fisher Scientific) according to the manufacturer’s protocols. To assess cytokine production following *Lm*OVA infection, splenocytes collected from infected mice at 7 dpi were stimulated with 1 µg ml^−1^ OVA_257–264_ peptide (Anaspec) and 50 U ml^−1^ IL-2 for 4 h at 37 °C. TIL were re-stimulated with PMA and ionomycin. GolgiStop protein transport inhibitor (BD Biosciences) was added at a 1:1,500 dilution during the last 2 h of incubation to block cytokine secretion. After stimulation, cells were stained with surface marker antibodies in staining buffer (PBS containing 2% FBS and 0.02% sodium azide) for 1 h at 4 °C. Cells were fixed and permeabilized using the Foxp3/Transcription Factor Staining Buffer Set (Thermo Fisher Scientific) at 4 °C for 1 h. Intracellular staining was performed by incubating cells with fluorescently labelled antibodies targeting intracellular markers for either 1 h or overnight at 4 °C. Flow cytometry data were acquired using a CytoFLEX (Beckman Coulter), Aurora Cytek or BD Accuri C6 Plus cytometer. Cell sorting was performed on an Astrios (Beckman Coulter) or BD FACSAria Fusion cell sorter. Data analysis was conducted using FlowJo software.

#### CITE-seq experimental protocol

C57BL/6J mice aged 10–12 weeks were allocated to either AL feeding or DR for 1 week before tumour cell injection. Following the feeding regimen, each mouse was subcutaneously injected with 5 × 10^5^ B16-OVA melanoma cells. At 13 dpi, tumours were dissected from the mice for further analysis. To prepare single-cell suspensions, excised tumours were mechanically dissociated and filtered sequentially through 100 µm and then 50 µm cell strainers. Throughout all procedures, cells were kept on ice to preserve viability. The resulting cell suspensions were counted, and cell viability was assessed by trypan blue exclusion. To prevent nonspecific antibody binding, cells were blocked with TruStain FcX Plus (BioLegend, 156603) in Cell Staining Buffer (CSB; BD Biosciences, 420201) for 10 min at 4 °C. Each tumour sample was then labelled with a specific antibody (Hashtags 1–8, product information in Key Resource Table) according to the manufacturer’s protocol, including appropriate incubation and washing steps.

After hashtag labelling, cells were stained with a panel of fluorescently labelled antibodies targeting surface markers to identify live CD45⁺ leucocytes while excluding red blood cells (Ter119⁻), tumour cells (CD105⁻) and dead cells (DAPI^+^). This staining facilitated the isolation of the desired cell population during flow cytometric sorting. Cells were sorted by flow cytometry, and live CD45⁺ cells were collected into tubes containing IMDM supplemented with 10% FBS. After sorting, cells were centrifuged and resuspended in CSB, and cell viability was reassessed. Based on cell counts, CD45⁺ cells were adjusted to a concentration of 1 × 10^6^ cells per 50 µl and blocked again with TruStain FcX Plus for 10 min at 4 °C. During this blocking step, TotalSeq C antibodies were prepared according to the manufacturer’s instructions (BioLegend). The TotalSeq C antibodies were then added to the CD45⁺ cells and incubated for 30 min at 4 °C to label cell surface proteins for subsequent analysis.

Following antibody staining, cells were washed three times with CSB to remove unbound antibodies. Cells were then resuspended in 1× PBS containing 0.04% pure BSA at an approximate concentration of 2,200 cells per µl. The prepared cell suspension was used for downstream library preparation and sequencing.

#### CITE-seq library preparation

Libraries were generated and sequenced by the VAI Genomics Core. Cells were processed with 10× Chromium Next GEM Single Cell 5ʹ GEM kit (v.1.1) according to the manufacturer’s instructions to target an output of 13,000 cells per sample using a 10× Genomics Chromium Controller. In brief, single-cell suspensions in PBS + 0.04% BSA were assessed for quantity and viability on the CytoFLEX S (Beckman Coulter), then 20,000 cells per sample were loaded onto the Chromium Controller. Single cells were captured in gel beads in emulsion, where they were lysed. Released RNA was barcoded and then converted to cDNA. Using beads, cDNA was separated from the hashtag oligonucleotides (HTO), and each was used to generate adaptor-ligated libraries. Quality and quantity of the finished gene expression and HTO libraries were assessed using a combination of Agilent DNA High Sensitivity Chip (Agilent Technologies) and QuantiFluor dsDNA System (Promega). Then, 2 × 100 bp, paired-end sequencing was performed on an Illumina NovaSeq 6000 sequencer using an S2, 200-cycle sequencing kit (v.1.5) to a minimum depth of 20 K reads per cell. Base calling was done by Illumina RTA3, and output was demultiplexed and converted to FastQ format with Cell Ranger (10× Genomics, v.3.1.0).

#### CITE-seq quality control and processing

Following library preparation, FastQ files were processed using Cell Ranger (v.7.0.1) to map and quantify expression profiles for both the transcriptome and epitopes. Single-cell 5′ paired-end sequencing chemistry was used, and the sequencing data were aligned to the mouse reference genome m10-2020-A. The resulting raw_feature_bc_matrix, containing both transcript-level and epitope quantifications, was imported into R using the Read10X function from the Seurat package (v.5.0.0). Initial data processing involved separating the gene expression (GEX) data, ADTs and HTOs, which was critical for downstream analysis. A Seurat object was created using the CreateSeuratObject function. Quality control and normalization steps were then performed: HTO data were normalized using the NormalizeData function with the method set to ‘CLR’ (centred log ratio transformation), and the HTODemux function was used to classify cells based on HTO signals into ‘singlets’, ‘doublets’ and ‘negative’ categories. Further quality control filtering was applied by retaining cells with ADT counts of at least 150, RNA counts of at least 200, an HTO classification of ‘singlet’ and mitochondrial gene expression percentage less than 20%. The GEX matrix was normalized using the SCTransform function (sctransform v.0.4.1), and ADT data were normalized using the NormalizeData function. Principal component analysis (PCA) was conducted on both RNA and ADT data using the RunPCA function, including all ADTs for dimensionality reduction.

To generate a joint uniform manifold approximation and projection (UMAP) embedding that integrates both GEX and ADT data, the FindMultiModalNeighbors function was used, followed by the RunUMAP function, resulting in a weighted nearest-neighbour UMAP. Cluster identification was achieved by integrating gene expression profiles, ADT data and gene expression density visualizations using the plot_density() function from the Nebulosa package (v.1.14.0). Details of cluster identification are provided in Supplementary Tables2 and 4 and Extended Data Figs.2 and 6. Statistical analyses for violin plots were performed using the Kruskal–Wallis test, followed by pairwise Wilcoxon comparison tests with Bonferroni *P* value adjustment. Sequencing data from the single-cell RNA sequencing (scRNA-seq) experiments have been deposited in the Gene Expression Omnibus (GEO) under accession number GSE267070.

#### RNA velocity estimation

To infer cellular trajectories from the scRNA-seq data, we performed RNA velocity analysis using the Python implementation of Velocyto (v.0.17.17), following previously described methods^45^. In brief, spliced and unspliced counts were generated using the velocyto run10x subcommand, outputting a loom file for importing into Python or R. Next, we used scvelo to infer RNA velocity vectors and projected them onto the UMAP embeddings generated above. This approach enabled us to determine the directional changes of cells between dietary conditions. Code used to make RNA velocity plots can be found at https://github.com/rgjcanada/DR_CITESEQ_2025.

#### Human TIL scRNA-seq

For analysis of the human scRNA-seq dataset^74^, count data were normalized and transformed through the SCT normalization method in Seurat, with 5,000 variable features retained for downstream dimension reduction techniques. Integration of data was performed on the patient level with canonical correlation analysis as the dimension reduction technique. Cells were clustered using the Louvain algorithm with multi-level refinement. PCA was performed, with the first 50 PCs used in UMAP generation. The data were subset to CD8^+^ T cells, which were identified using the labels provided in a prior work^75^ and confirmed by singleR using the ImmGen database^76^ and cell type annotation with the ProjectTILs T cell atlas^77^. These CD8^+^ T cells were subsequently normalized through the SCT method, with 3,000 variable features retained. Owing to the low number of cells on a per-patient level, Harmony was used to integrate the data at the patient level rather than Seurat^78^. PCA and UMAP dimension reduction were performed as above for clustering into distinct groups, Teff_ITGB1, Teff_TYROBP, Teff_TRIM34, TCM, Trm_TM, Trm_TMEM2, Trm_BAG3, Trm_ZNF20, IEL, Tex_MKI67 and Tex_CLNK, based on previously defined markers^79^. To simplify the analysis, we combined Teff_ITGB1, Teff_TYROBP and Teff_TRIM34 into the ‘T effector (T_eff_)’ cluster, while grouping TCM, Trm_TM, Trm_TMEM2, Trm_BAG3, Trm_ZNF20 and IEL into the ‘T memory (T_mem_)’ cluster. Additionally, Tex_MKI67 and Tex_CLNK were combined into the ‘T exhausted (T_ex_)’ cluster.

#### Sample preparation for proteomics

Samples were processed using the EasyPep Mini MS Sample Prep Kit (Thermo Fisher Scientific, A40006) according to the manufacturer’s instructions. In brief, cells were resuspended in 50 µl of lysis solution and proteins were quantified using the Pierce BCA Protein Assay Kit (Thermo Fisher Scientific, 23227) following the vendor-supplied protocol. Plates were read at an absorbance of 562 nm using the Synergy LX Multi-Mode Reader, and Gen5 software was used for data analysis (BioTek/Agilent). Polynomial regression was used in the Gen5 software to calculate protein concentrations from a protein standard curve after an average blank absorbance subtraction. Then, 25 µg of protein per sample was reduced and alkylated at 95 °C for 10 min, and samples were digested overnight with Trypsin/Lys-C at 30 °C at a ratio of 10:1 (protein:enzyme (w/w)). Resulting peptides were cleaned with the supplied peptide clean-up columns in the EasyPep mini kit and then dried down in a Genevac SpeedVac before resuspension for instrument analysis. Samples were resuspended in 12.5 µl 0.1% formic acid (Fisher Scientific, LS118-1) and diluted 1:1 with 6 µl of 0.1% trifluoroacetic acid (Fisher Scientific, LS119-500) in autosampler vials.

#### Data-independent acquisition LC–tandem MS proteomics

Data-independent acquisition analyses were performed on an Orbitrap Eclipse coupled to a Vanquish Neo system (Thermo Fisher Scientific) with a FAIMS Pro source (Thermo Fisher Scientific) located between the nanoESI source and the mass spectrometer. A total of 2 μg of digested peptides were separated on a nano capillary column (20 cm × 75 μm inner diameter, 365 μm outer diameter, 1.7 μm C18; CoAnn Technologies, HEB07502001718IWF) at 300 nl min^−1^. Mobile phase A consisted of LC–MS-grade H_2_O (Fisher Scientific, LS118-500), mobile phase B consisted of 20% LC–MS-grade H_2_O and 80% LC–MS-grade acetonitrile (Fisher Scientific, LS122500), both containing 0.1% formic acid. The LC gradient was 1% B to 24% B in 110 min; 85% B in 5 min; and 98% B for 5 min; with a total gradient length of 120 min. The column temperature was kept constant at 50 °C using a customized column heater (Phoenix S&T). For FAIMS, the selected compensation voltage was applied (−40 V, −55 V and −70 V) throughout the LC–MS/MS runs. Full MS spectra were collected at 120,000 resolution (full width half-maximum; FWHM), and MS2 spectra at 30,000 resolution (FWHM). Both the standard automatic gain control target and the automatic maximum injection time were selected. A precursor range of 380–985 *m*/*z* was set for MS2 scans, and an isolation window of 50 *m*/*z* was chosen with a 1 *m*/*z* overlap for each scan cycle; 32% HCD collision energy was used for MS2 fragmentation.

Data-independent acquisition data were processed in Spectronaut (v.19; Biognosys) using direct data-independent acquisition. Data were searched against the *Mus musculus* proteome. The manufacturer’s default parameters were used. In brief, trypsin/P was set as the digestion enzyme, and two missed cleavages were allowed. Cysteine carbamidomethylation was set as a fixed modification, and methionine oxidation and protein amino-terminal acetylation as variable modifications. Identification was performed using a 1% *q* value cutoff on precursor and protein levels. Both peptide precursors and protein false discovery rate (FDR) were controlled at 1%. Ion chromatograms of fragment ions were used for quantification. For each targeted ion, the area under the curve between the XIC peak boundaries was calculated.

#### Proteomic data processing and GSEA

Proteomic values were imported into R from an Excel file and processed with dplyr and readxl. Replicate columns for each condition (AL-WT, AL-DKO, DR-WT, DR-DKO) were coerced to numeric and averaged to obtain per-protein means. Differential abundance was calculated as log_2_(fold change) (for example, DR-WT / AL-WT), and Welch’s *t*-tests were performed between the corresponding replicate groups to obtain *P* values. Proteins lacking sufficient replicate measurements were excluded.

For GSEA, proteins were mapped to gene symbols (duplicated symbols collapsed by the entry with the largest absolute log_2_(fold change). A pre-ranked vector was generated using the sign of the log2(fold change) multiplied by −log_10_(*P* value) (equivalent results were obtained using log_2_(fold change) alone). Mouse gene sets were retrieved from MSigDB via msigdbr (Hallmark ‘H’, curated ‘C2’ and Gene Ontology ‘C5’ collections); sets with <15 or >500 genes were discarded. Enrichment analysis was performed with fgsea (10,000 permutations, default weighting), returning normalized enrichment scores, nominal *P* values and FDR-adjusted *q* values (Benjamini–Hochberg). Only pathways with an FDR *q* < 0.05 were considered significant and are displayed in the figures. Bar plots of normalized enrichment scores and volcano plots were generated in ggplot2 and EnhancedVolcano, respectively.

#### Promethion metabolic cages

Mice were individually housed in Promethion metabolic cages (Sable Systems International) for comprehensive metabolic phenotyping. Before data collection, animals underwent a 24 h acclimation period within the cages to minimize stress and ensure steady-state measurements. Following acclimation, continuous monitoring was performed over a desired period under controlled environmental conditions (temperature, humidity and a 12–12h light–dark cycle) as specified by the manufacturer’s guidelines.

The Promethion system simultaneously recorded oxygen consumption, carbon dioxide production and energy expenditure, as well as food and water intake and locomotor activity. Calibration of the sensors and verification of system performance were conducted in strict accordance with Sable Systems International’s operating protocols. Data acquisition and subsequent analysis were carried out using the proprietary Promethion analysis software, ensuring that all measurements met the company’s quality control standards.

#### EchoMRI body composition analysis

Body composition was measured using an EchoMRI-100 on conscious mice. Before each session, the instrument was calibrated with manufacturer-provided oil standards. Individual mice were weighed, placed in a ventilated acrylic restraining tube with an internal insert to minimize movement and scanned (standard mode; water stage on–off as indicated in figure legends). Each scan yielded fat mass, lean mass, free water and total water; values were exported directly from the EchoMRI software for downstream analysis. Restraining tubes were disinfected between animals with 70% ethanol or facility-approved disinfectant.

#### CD8^+^ DAB staining quantification

Full-resolution SVS images of HDAB-stained tissue sections were imported into QuPath (v.0.5.1)^80^ for analysis. For all tissues to be analysed, a region of interest contouring the tissue was created, using the SAM plugin (v.0.6.0) for QuPath^81,82^ with the following settings: vit_h (huge) model, foreground with rectangle draw prompt and single mask output on live mode. To detect nuclei, an average of the deconvolved haematoxylin, DAB and residual channels was generated to create a fluorescence-like single-channel image using a custom script (hosted on https://github.com/vaioic). Random regions from the samples were used for CellPose’s human-in-the-loop training based on the livecell_cp3 model to improve the detection of nuclei containing DAB staining^83–85^. Detections were then generated on the average channel image using the custom-trained CellPose-based model using the CellPose plugin for QuPath (v.9.0) (https://github.com/BIOP/qupath-extension-cellpose). The detections and tissue outline were exported as an object GeoJSON and imported to the original HDAB-stained image. Detection measurements were generated with QuPath’s built-in Add Intensity Features with the following settings: pixel size, 0.5 µm; region, region of interest; tile diameter, 0; channels, haematoxylin, DAB; basic features, mean, standard deviation, minimum, maximum and median. Nuclei were then classified as positive or negative for DAB staining using the mean DAB signal at a threshold of 0.117. A pixel thresholder was used to measure the area covered by DAB staining. A training image generated from an equal number and size of random rectangles from each image was used for parameter setting. QuPath’s Create Thresholder was used with the following settings: resolution, very high (1.01 µm per pixel); channel, DAB; prefilter, Gaussian; smoothing sigma, 1; threshold, 0.25. The measure option was then run on the tissue outline to quantify the area of the tissue covered by DAB staining.

#### Statistics and data reproducibility

Data are presented as means ± s.d. for technical replicates or means ± s.e.m. for biological replicates, as indicated in figure legends. Statistical analyses were performed using GraphPad Prism (GraphPad Software, v.10.4.1) unless otherwise noted. Comparisons between two groups were conducted using unpaired, two-tailed Student’s *t*-tests. For comparisons involving more than two groups, one-way or two-way ANOVA with post hoc multiple-comparison corrections (Tukey’s or Sidak’s test, as appropriate) were applied. Kaplan–Meier survival curves were compared using the log-rank (Mantel–Cox) test, with Bonferroni correction applied for multiple comparisons when indicated. Data distribution was assumed to be normal. Statistical significance was defined as *P* < 0.05, with significance levels represented as *P < 0.05, **P < 0.01, ***P < 0.001 and ****P < 0.0001. The statistical tests used for each dataset are specified in the corresponding figure legends when deviating from the above.

No statistical method was used to predetermine sample size, but our sample sizes are similar to those reported in previous publications. All data were included in the analyses. In rare cases for which data were excluded, exclusion occurred because of animal health concerns (for example, sickness or tissue necrosis) or based on statistical outlier identification using standard outlier exclusion tests. Investigators were blinded to outcome assessment but not to experimental allocation during data collection. All experiments were independently repeated as described in the figure legends, and similar results were obtained, confirming reproducibility.

### Declaration of generative AI and AI-assisted technologies

During the preparation of this work, the author(s) used OpenAI’s ChatGPT to review grammar, spelling and refine text style. Following its use, the author(s) thoroughly reviewed and edited the content as needed and assume full responsibility for the final version of the publication.

### Reporting summary

Further information on research design is available in the Nature Portfolio Reporting Summary linked to this article.

## Supplementary information

Supplementary Information Key resources table

Reporting summary

Supplementary Dataset 2 5010 diet makeup

Supplementary Dataset 3 CD45^+^ cluster information

Supplementary Dataset 4 Spleen immunophenotyping

Supplementary Dataset 5 Proteomic results

Supplementary Dataset 6 CD8^+^ cluster information

Supplementary Dataset 7 Tumour lipidomics results

Supplementary Dataset 8 Tumour metabolomics results

Supplementary Dataset 9 DR tracing results

Supplementary Dataset 10 Chronic stim results

## Source data

Source Data Fig. 1 Raw source data Fig. 1

Source Data Fig. 2 Raw source data Fig. 2

Source Data Fig. 3 Raw source data Fig. 3

Source Data Fig. 4 Raw source data Fig. 4

Source Data Fig. 5 Raw source data Fig. 5

Source Data Fig. 6 Raw source data Fig. 6

Source Data Fig. 7 Raw source data Fig. 7

Source Data Fig. 8 Unprocessed western blots

Source Data Extended Data Fig./Table 1 Statistical source data

Source Data Extended Data Fig./Table 2 Statistical source data

Source Data Extended Data Fig./Table 3 Statistical source data

Source Data Extended Data Fig./Table 4 Statistical source data

Source Data Extended Data Fig./Table 5 Statistical source data

Source Data Extended Data Fig./Table 6 Statistical source data

Source Data Extended Data Fig./Table 7 Statistical source data

Source Data Extended Data Fig./Table 8 Statistical source data

Source Data Extended Data Fig./Table 9 Statistical source data

Source Data Extended Data Fig./Table 10 Statistical source data

## Data Availability

All unique and stable reagents generated in this study are available from the Lead Contact upon completion of a Materials Transfer Agreement. CITE-seq data of mouse TILs have been deposited in the National Center for Biotechnology Information Gene Expression Omnibus (NCBI GEO) under accession number GSE267070. Human scRNA-seq data of TILs used in this research are available at NCBI GEO under accession number GSE146771. All other data supporting the findings of this study are available within the article and its online supplementary material. Bioenergetics data were analysed using protocols developed by Mookerjee and Brand, available for download at https://russelljoneslab.vai.org. Source data are provided with this paper.

## Code availability

R code used for data processing is available at our GitHub repository (https://github.com/rgjcanada/DR_CITESEQ_2025.git). Processed data files (RDS files) curated from the data processing can be found at Zenodo (10.5281/zenodo.13920173)^86^.
