# Supplementary material for: Dietary restriction reprograms CD8+ T cell fate to enhance anti-tumour immunity and immunotherapy responses
Source: Nat Metab. 2025 Dec 9;7(12):2489–509. doi: 10.1038/s42255-025-01415-6 (PMC12727518; doi:10.1038/s42255-025-01415-6)
Supplement: Supplementary file 1 — Key resources table [file 42255_2025_1415_MOESM1_ESM.pdf]

nature metabolism

Supplementary information https://doi.org/10.1038/s42255-025-01415-6

Dietary restriction reprograms $CD8^+$ T cell fate to enhance anti-tumour immunity and immunotherapy responses

In the format provided by the authors and unedited

Key Resources Table

REAGENT or RESOURCE	SOURCE	IDENTIFIER
**Antibodies**		
Hamster monoclonal anti-mouse CD3e (145-2C11)	Thermo Fisher Scientific	Cat# 16-0031-82; RRID: AB_468847
Hamster monoclonal anti-mouse CD28 (37.51)	Thermo Fisher Scientific	Cat# 16-0281-86; RRID: AB_468923
Rat monoclonal anti-mouse CD8a (53-6.7), BUV395	BD Biosciences	Cat# 563786; RRID: AB_2732919
Rat monoclonal anti-mouse CD8a (53-6.7), BUV737	BD Biosciences	Cat# 612759; RRID: AB_2870090
Rat monoclonal anti-mouse CD44 (IM7), BUV805	BD Biosciences	Cat# 741921; RRID: AB_2871234
Mouse monoclonal anti-mouse NK-1.1 (PK136), Brilliant Violet 605	BioLegend	Cat# 108739; RRID: AB_2562273
Rat monoclonal anti-mouse CD127 (A7R34), Brilliant Violet 785	BioLegend	Cat# 135037; RRID: AB_2565269
Rat monoclonal anti-mouse CD3 (17A2), FITC	Thermo Fisher Scientific	Cat# 11-0032-82; RRID: AB_2572431
Rat monoclonal anti-mouse CD4 (RM4-5), FITC	Thermo Fisher Scientific	Cat# 11-0042-82; RRID: AB_464896
Hamster monoclonal anti-mouse KLRG1 (2F1), Alexa Fluor 532	Thermo Fisher Scientific	Cat# 58-5893-82; RRID: AB_2815282
Rat monoclonal anti-mouse CD19 (1D3), PE	BioLegend	Cat# 152408; RRID: AB_2629817
Mouse monoclonal anti-mouse CD90.1/Thy1.1 (HIS51), PE	Thermo Fisher Scientific	Cat# 12-0900-81; RRID: AB_465773

Mouse monoclonal anti-human/mouse Granzyme B (QA16A02), PE/Dazzle 594	BioLegend	Cat# 372216; RRID: AB_2728383
Rat monoclonal anti-mouse CD8a (53-6.7), PE-Cyanine7	Thermo Fisher Scientific	Cat# 25-0081-82; RRID: AB_469584
Rat monoclonal anti-mouse TNF-alpha (MP6-XT22), PE-Cyanine7	Thermo Fisher Scientific	Cat# 25-7321-82; RRID: AB_11042728
Rat monoclonal anti-mouse CD4 (RM4-5), APC	Thermo Fisher Scientific	Cat# 17-0042-82; RRID: AB_469323
Rat monoclonal anti-mouse IFN-gamma (XMG1.2), APC	Thermo Fisher Scientific	Cat# 17-7311-82; RRID: AB_469504
Rabbit polyclonal anti-β-ACTIN	Cell Signaling Technology	Cat# 4967; RRID: AB_330288
Goat anti-rabbit IgG, HRP-conjugated	Cell Signaling Technology	Cat# 7074; RRID: AB_2099233
BDH1 antibody	Proteintech	Cat# 15417-1-AP, RRID:AB_2274683
BDH1 antibody	Proteintech	Cat# 67448-1-Ig, RRID:AB_2882682
OXCT1 antibody	Proteintech	Cat# 12175-1-AP, RRID:AB_2157444
Alexa Fluor(R) 700 anti-mouse CX3CR1	BioLegend	Cat# 149036, RRID:AB_2629606
CD62L (L-Selectin)	BD Biosciences	Cat# 740218, RRID:AB_2739966
Brilliant Violet 711(TM) anti-mouse CD69	BioLegend	Cat# 104537, RRID:AB_2566120
Pacific Blue(TM) anti-mouse Ly108	BioLegend	Cat# 134608, RRID:AB_2188093
Brilliant Violet 605(TM) anti-mouse CD279 (PD-1)	BioLegend	Cat# 135219, RRID:AB_11125371
CD366 (TIM3) Monoclonal Antibody (RMT3-23), APC, eBioscience	Thermo Fisher Scientific	Cat# 17-5870-82, RRID:AB_2688131
IFN gamma Monoclonal Antibody (XMG1.2), APC, eBioscience	Thermo Fisher Scientific	Cat# 17-7311-82, RRID:AB_469504

TNF alpha Monoclonal Antibody (MP6-XT22), PE-Cyanine7, eBioscience	Thermo Fisher Scientific	Cat# 25-7321-82, RRID:AB_11042728
TOX Antibody, anti-human/mouse, PE, REAfinity™	Miltenyi Biotec	Cat# 130-120-785, RRID:AB_2801785
TCF1/TCF7 (C63D9) Rabbit mAb (Alexa Fluor® 647 Conjugate)	Cell Signaling Technology	Cat# 6709, RRID:AB_2797631
Brilliant Violet 605(TM) anti-T-bet	BioLegend	Cat# 644817, RRID:AB_11219388
Goat polyclonal anti-Armenian hamster IgG (H+L), secondary antibody, biotin-conjugated	Thermo Fisher Scientific	Cat# 13-4113-85; RRID: AB_466651
TotalSeq(TM)-CMouse Universal Cocktail, V1.0	BioLegend	Cat# 199903, RRID:AB_2924498
TotalSeq(TM)-C0301 anti-mouse Hashtag 1	BioLegend	Cat# 155861<br>RRID:AB_2800693
TotalSeq(TM)-C0302 anti-mouse Hashtag 2	BioLegend	Cat# 155863, RRID:AB_2800694
TotalSeq(TM)-C0303 anti-mouse Hashtag 3	BioLegend	Cat# 155865, RRID:AB_2800695
TotalSeq(TM)-C0304 anti-mouse Hashtag 4	BioLegend	Cat# 155867, RRID:AB_2800696
TotalSeq(TM)-C0305 anti-mouse Hashtag 5	BioLegend	Cat# 155869, RRID:AB_2800697
TotalSeq(TM)-C0306 anti-mouse Hashtag 6	BioLegend	Cat# 155871, RRID:AB_2819910
TotalSeq(TM)-C0307 anti-mouse Hashtag 7	BioLegend	Cat# 155873, RRID:AB_2819911
TotalSeq(TM)-C0308 anti-mouse Hashtag 8	BioLegend	Cat# 155875, RRID:AB_2819912
TruStain FcX(TM) PLUS (anti-mouse CD16/32)	BioLegend	Cat# 156603, RRID:AB_2783137
Cell Staining Buffer	BioLegend	Cat# 420201
APC anti-mouse CD45	BioLegend	Cat# 103111, RRID:AB_312976

FITC anti-mouse TER-119/Erythroid Cells	BioLegend	Cat# 116206, RRID:AB_313707
CD105 (Endoglin) Monoclonal Antibody (SN6), PE, eBioscience	Thermo Fisher Scientific	Cat# 12-1057-42, RRID:AB_1311123
InVivoPlus polyclonal Armenian hamster IgG	Bio X Cell	Cat# BP0091, RRID:AB_1107773
InVivoPlus anti-mouse PD-1 (CD279)	Bio X Cell	Cat# BP0033-2, RRID:AB_1107747
**Bacterial and Virus Strains**		
Attenuated (ΔactA) *Lm*OVA	John Harty	Haring *et al.*[66]
**Chemicals, Peptides, and Recombinant Proteins**		
DMEM with 4.5 g/L glucose and L-glutamine, without sodium pyruvate	Wisent Inc.	Cat# 319-015-CL
IMDM with L-glutamine & 25 mM HEPES	Wisent Inc.	Cat# 319-105-CL
Van Andel Institute-modified IMDM (VIM)	Custom	Kaymak *et al.*[21]
Seahorse XF base medium, without phenol red	Agilent Technologies	Part# 103335-100
Nu-Serum IV Culture Supplement	Corning	Cat# 355504; Lot# 2080003
Fetal bovine serum (FBS), heat-inactivated	Corning	Cat# 35-016-CV; Lot# 16821001
Fetal bovine serum (FBS), dialyzed	Corning	Cat# 35-071-CV; Lot# 35071105
Pen Strep (5,000 U/mL penicillin and 5,000 μg/mL streptomycin)	Gibco	Cat# 15070063
2-mercaptoethanol (55 mM; 1000x)	Gibco	Cat# 21985023
Recombinant murine IL-2	Peprotech	Cat# 212-12
Recombinant human IL-2	Peprotech	Cat# 200-02
ImmunoCult Human CD3/CD28 T Cell Activator	StemCell Technologies	Cat# 10971
D-Glucose [U-$^{13}C_6$]	Cambridge Isotopes	Cat# CLM-1396
Sodium D-3-hydroxybutyrate (2,4-$^{13}C_2$)	Cambridge Isotopes	Cat# CLM-3706-1

Sodium D-3-hydroxybutyrate ($^{13}C_4$)	Cambridge Isotopes	Cat# CLM-3853-PK
D-Glucose	Sigma-Aldrich	Cat# G8270
L-Glutamine	Sigma-Aldrich	Cat# G3126
Fixable Viability Dye eFluor 506	Thermo Fisher Scientific	Cat# 65-0866-14
Fixable Viability Dye eFluor 780	Thermo Fisher Scientific	Cat# 65-0865-14
Violet Proliferation Dye 450	BD Biosciences	Cat# 562158
BD GolgiStop	BD Biosciences	Cat# 51-2092KZ
Foxp3/Transcription Factor Staining Buffer Set	Thermo Fisher Scientific	Cat# 00-5523-00
$OVA_{257-264}$ (SIINFEKL) peptide	AnaSpec	Cat# AS-60193-1
cOmplete, EDTA-free (protease inhibitor cocktail tablets)	Roche	Cat# 11873580001
PhosSTOP (phosphatase inhibitor cocktail tablets)	Roche	Cat# 4906845001
Monensin sodium salt	Sigma-Aldrich	Cat# M5273
Streptavidin, Alexa Fluor 647	Thermo Fisher Scientific	Cat# S21374
Phorbol 12-myristate 13-acetate (PMA)	Millipore Sigma	Cat# P1585
Ionomycin calcium salt	Millipore Sigma	Cat# I0634
**Critical Commercial Assays**		
EasySep Mouse T cell Isolation Kit	StemCell Technologies	Cat# 19851
EasySep Mouse CD8$^+$ T cell Isolation Kit	StemCell Technologies	Cat# 19853
EasySep Mouse CD90.1 Positive Selection Kit	StemCell Technologies	Cat# 18958
Seahorse XFe96 FluxPak	Agilent Technologies	Part# 102416-100
Seahorse XF Cell Mito Stress Test Kit	Agilent Technologies	Part# 103015-100

Pierce BCA Protein Assay Kit	Thermo Fisher Scientific	Cat# 23225
Pierce Rapid Gold BCA Protein Assay Kit	Thermo Fisher Scientific	Cat# A53227
**Deposited data**		
CITE-Sequencing FASTQ	This manuscript	NCBI GEO: GSE267070
RDS Files (Processed data)	This manuscript	10.5281/zenodo.13920172
Human Single Cell RNA Sequencing	Zhang L *et al.*[67]	NCBI GEO: GSE146771
**Experimental Models: Cell Lines**		
293T cells	ATCC	CRL-3216
MC38-OVA-tdTomato cells		Luda *et al.*[22]
B16-OVA cells		Cordeiro *et al.*[68]
EO-771 cells	ATCC	CRL-3461
**Experimental Models: Organisms/Strains**		
C57BL/6J mice	The Jackson Laboratory	RRID: IMSR_JAX:000664
C57BL/6-Tg(TcraTcrb)1100Mjb/J (OT-I) mice	The Jackson Laboratory	RRID: IMSR_JAX:003831
B6.PL-*Thy1*^{a}/CyJ (Thy1.1) mice	The Jackson Laboratory	RRID: IMSR_JAX:000406
B6.Cg-Tg(Cd4-cre)1Cwi/BfluJ (*Cd4-Cre*) mice	The Jackson Laboratory	RRID: IMSR_JAX:022071
Bdh1$^{fl/fl}$/*Oxct1*$^{fl/fl}$*Cd4-Cre*	This paper	
Bdh1$^{fl/fl}$/*Oxct1*$^{fl/fl}$*Cd4-Cre* OT-I	This paper	
**Oligonucleotides**		
Bdh1 genotyping primers:<br>Forward:<br>TGCAGGAATCAGTGCTCTCTCCTAGCA<br>Reverse: GGT GTC AGG GCT GAA GGA TG	This paper	Custom
Oxct1 genotyping primers:<br>Forward:	This paper	Custom

TATGGGACTCTGGTACAGGAAG<br>Reverse:<br>TGCTGACCGTTAAACTCCCTC		
**Software and Algorithms**		
Adobe Illustrator v28.1	Adobe	adobe.com/products/illustrator.html
Fiji v2.15.1		imagej.net/software/fiji
FlowJo v9.9.5	FlowJo LLC	flowjo.com
GraphPad Prism v9-10	GraphPad Software	graphpad.com
IncuCyte v2022A Rev1	Sartorius	sartorius.com/en
R v4.4.0		cran.r-project.org
Python		V3.12.0
