# Supplementary material for: Dietary restriction reprograms CD8+ T cell fate to enhance anti-tumour immunity and immunotherapy responses
Source: Nat Metab. 2025 Dec 9;7(12):2489–509. doi: 10.1038/s42255-025-01415-6 (PMC12727518; doi:10.1038/s42255-025-01415-6)
Supplement: Supplementary file 2 — Reporting summary [file 42255_2025_1415_MOESM2_ESM.pdf]

nature portfolio

Corresponding author(s): Brandon M. Oswald, Lisa M. DeCamp, Joseph Longo, Michael S. Dahabieh, Nicholas Bunda, Benjamin K. Johnson, McLane J. Watson, Shixin Ma, Samuel E J. Preston, Ryan D. Sheldon, Michael P. Vincent, Abigail E Ellis, Molly T. Soper-Hopper, Christine Isaguirre, Dahlya Kamarudin, Hui Shen, Kelsey S. Williams, Peter A. Crawford, Susan Kaech, H. Josh Jang, Evan C. Lien ,Connie M. Krawczyk, and Russell G. Jones

Last updated by author(s): Oct 22, 2025

Reporting Summary

Nature Portfolio wishes to improve the reproducibility of the work that we publish. This form provides structure for consistency and transparency in reporting. For further information on Nature Portfolio policies, see our Editorial Policies and the Editorial Policy Checklist.

Statistics

For all statistical analyses, confirm that the following items are present in the figure legend, table legend, main text, or Methods section.

n/a	Confirmed	
☐	☒	The exact sample size (*n*) for each experimental group/condition, given as a discrete number and unit of measurement
☐	☒	A statement on whether measurements were taken from distinct samples or whether the same sample was measured repeatedly
☐	☒	The statistical test(s) used AND whether they are one- or two-sided<br>*Only common tests should be described solely by name; describe more complex techniques in the Methods section.*
☐	☒	A description of all covariates tested
☐	☒	A description of any assumptions or corrections, such as tests of normality and adjustment for multiple comparisons
☐	☒	A full description of the statistical parameters including central tendency (e.g. means) or other basic estimates (e.g. regression coefficient) AND variation (e.g. standard deviation) or associated estimates of uncertainty (e.g. confidence intervals)
☐	☒	For null hypothesis testing, the test statistic (e.g. *F*, *t*, *r*) with confidence intervals, effect sizes, degrees of freedom and *P* value noted<br>*Give P values as exact values whenever suitable.*
☒	☐	For Bayesian analysis, information on the choice of priors and Markov chain Monte Carlo settings
☒	☐	For hierarchical and complex designs, identification of the appropriate level for tests and full reporting of outcomes
☒	☐	Estimates of effect sizes (e.g. Cohen's *d*, Pearson's *r*), indicating how they were calculated

Our web collection on statistics for biologists contains articles on many of the points above.

Software and code

Policy information about availability of computer code

Data collection	Flow cytometry data was collected using SpectroFlo software (version 3.0.3) from Cytek.<br>Mass spectrometry peak picking and integration were completed in Skyline (v23.3) using an in-house curated compound data base from analytical standards.<br>Lipidomics samples were analyzed with a Thermo Vanquish dual liquid chromatography system.<br>Libraries were generated and sequenced by the Van Andel Institute Genomics Core.<br>Cells were processed with 10X Chromium Next GEM Single Cell 5′ GEM kit v1.1 (10X Genomics, Pleasanton, CA) according to the manufacturer's instructions.
Data analysis	Flow cytometry analysis was conducted using FlowJo software (version 10.10.0).<br><br>Mass spectrometry (metabolomics) natural abundance correction was performed using IsoCorrectoR.<br>Proteomics DIA data were processed in Spectronaut (version 18, Biognosys, Switzerland) using directDIA'" analysis.<br><br>Data analysis and graphing was conducted using Excel (Microsoft) and Prism software.

FastQ files were processed using Cell Ranger version 7.0.1
to map and quantify expression profiles for both the transcriptome and epitopes. Single-cell 5′ paired-end sequencing chemistry was employed, and the sequencing data were aligned to the mouse reference genome m10-2020-A

For manuscripts utilizing custom algorithms or software that are central to the research but not yet described in published literature, software must be made available to editors and reviewers. We strongly encourage code deposition in a community repository (e.g. GitHub). See the Nature Portfolio guidelines for submitting code & software for further information.

Data

Policy information about availability of data

All manuscripts must include a data availability statement. This statement should provide the following information, where applicable:

- Accession codes, unique identifiers, or web links for publicly available datasets
- A description of any restrictions on data availability
- For clinical datasets or third party data, please ensure that the statement adheres to our policy

All unique and stable reagents generated in this study are available from the Lead Contact upon completion of a Materials Transfer Agreement. CITE-sequencing data of mouse tumor-infiltrating lymphocytes have been deposited in the National Center for Biotechnology Information Gene Expression Omnibus (NCBI GEO) under accession number GSE267070. Human single-cell RNA-sequencing data of tumor-infiltrating lymphocytes used in this research are available at NCBI GEO under accession number GSE146771. All other data supporting the findings of this study are available within the article and its online supplementary material. Bioenergetics data were analyzed using protocols developed by Mookerjee and Brand, available for download at https://russelljoneslab.vai.org. R code used for data processing is available at our GitHub repository (https://github.com/rgjcanada/DR_CITESEQ_2025.git). Processed data files (RDS files) curated from the data processing can be found at https://doi.org/10.5281/zenodo.13920173.

Research involving human participants, their data, or biological material

Policy information about studies with human participants or human data. See also policy information about sex, gender (identity/presentation), and sexual orientation and race, ethnicity and racism.

Reporting on sex and gender	n/a
Reporting on race, ethnicity, or other socially relevant groupings	n/a
Population characteristics	n/a
Recruitment	n/a
Ethics oversight	n/a

Note that full information on the approval of the study protocol must also be provided in the manuscript.

Field-specific reporting

Please select the one below that is the best fit for your research. If you are not sure, read the appropriate sections before making your selection.

☒ Life sciences ☐ Behavioural & social sciences ☐ Ecological, evolutionary & environmental sciences

For a reference copy of the document with all sections, see nature.com/documents/nr-reporting-summary-flat.pdf

Life sciences study design

All studies must disclose on these points even when the disclosure is negative.

Sample size	All in vivo experiments included between 5 and 20 mice per group, depending on the model and endpoint, and were independently reproduced 2–3 times. No statistical methods were used to predetermine sample sizes; however, our group sizes are consistent with those reported in similar studies. For mouse studies, data distribution was assumed to be normal but this assumption was not formally tested.
Data exclusions	Data were excluded only when mice were deemed unhealthy (e.g., severe illness, necrotic tumors, or unsafe conditions) or when exclusion was supported by clear statistical rationale (e.g., technical outliers identified by predefined criteria).
Replication	Experiments were successfully replicated 2-3 times. All attempts at replication were successful
Randomization	Mice were randomly assigned to experimental groups using cage-based randomization. Both male and female mice were used in most experiments to minimize sex bias. In one experiment, (Fig. 7G) only female mice were used, as noted in the corresponding figure legends, due to established tumor model and housing constraints. Investigators were blinded to group allocation during data collection and analysis whenever feasible.

Blinding | Animal experiments were initiated by one lab member, who generated a key for genotypes and treatment groups. A second lab member conducted experiments. After completion of experiments the answer key was unblinded.

Reporting for specific materials, systems and methods

We require information from authors about some types of materials, experimental systems and methods used in many studies. Here, indicate whether each material, system or method listed is relevant to your study. If you are not sure if a list item applies to your research, read the appropriate section before selecting a response.

Materials & experimental systems

n/a	Involved in the study
☐	☒ Antibodies
☐	☒ Eukaryotic cell lines
☒	☐ Palaeontology and archaeology
☐	☒ Animals and other organisms
☒	☐ Clinical data
☒	☐ Dual use research of concern
☒	☐ Plants

Methods

n/a	Involved in the study
☒	☐ ChIP-seq
☐	☒ Flow cytometry
☒	☐ MRI-based neuroimaging

Antibodies

Antibodies used | Immunoblotting was performed using primary antibodies against BDH1 (Proteintech, Cat# 15417-1-AP and Cat# 67448-1-Ig), OXCT1 (Proteintech, Cat# 12175-1-AP), and β-ACTIN (Cell Signaling Technology, Cat# 4967), with HRP-conjugated secondary goat anti-rabbit IgG (Cell Signaling Technology, Cat# 7074).

Flow cytometry employed a comprehensive panel of fluorescently labeled antibodies, including CD3ε (145-2C11, Thermo Fisher Scientific, Cat# 16-0031-82), CD28 (37.51, Thermo Fisher Scientific, Cat# 16-0281-86), CD8a (53-6.7, BUV395, BD Biosciences, Cat# 563786; BUV737, BD Biosciences, Cat# 612759; PE-Cy7, Thermo Fisher Scientific, Cat# 25-0081-82), CD44 (IM7, BUV805, BD Biosciences, Cat# 741921), NK-1.1 (PK136, Brilliant Violet 605, BioLegend, Cat# 108739), CD127 (A7R34, BV785, BioLegend, Cat# 135037), KLRG1 (2F1, AF532, Thermo Fisher Scientific, Cat# 58-5893-82), CD4 (RM4-5, FITC, Thermo Fisher Scientific, Cat# 11-0042-82; APC, Cat# 17-0042-82), CD90.1/Thy1.1 (HIS51, PE, Thermo Fisher Scientific, Cat# 12-0900-81), Granzyme-B (QA16A02, PE/Dazzle594, BioLegend, Cat# 372216), CX3CR1 (AF700, BioLegend, Cat# 149036), CD62L (L-Selectin, BD Biosciences, Cat# 740218), CD69 (BV711, BioLegend, Cat# 104537), Ly108 (Pacific Blue, BioLegend, Cat# 134608), PD-1 (CD279, BV605, BioLegend, Cat# 135219), TIM-3 (RMT3-23, APC, eBioscience, Thermo Fisher Scientific, Cat# 17-5870-82), IFN-γ (XMG1.2, APC, eBioscience, Thermo Fisher Scientific, Cat# 17-7311-82), TNF-α (MP6-XT22, PE-Cy7, eBioscience, Thermo Fisher Scientific, Cat# 25-7321-82), TOX (REA473, PE, Miltenyi Biotec, Cat# 130-120-785), TCF1/TCF7 (C63D9, Alexa Fluor 647, Cell Signaling Technology, Cat# 6709), and T-bet (BV605, BioLegend, Cat# 644817). Staining was performed as per manufacturer's instructions, and data were acquired on CytoFLEX, Aurora Cytek, or BD Accuri C6 Plus instruments.

Validation | All antibodies were validated on the manufacturer's website. See product number and description.

Eukaryotic cell lines

Policy information about cell lines and Sex and Gender in Research

Cell line source(s) | 293T cells used for retrovirus production were obtained from ATCC (CRL-3216). EO-771 breast cancer cells were obtained from ATCC (CRL-3461). B16-F10 melanoma cells expressing OVA (B16-OVA) were provided as previously described by Cordeiro et al., and MC38-OVA-tdTomato cells were provided by Luda et al. All cell lines were authenticated by the respective suppliers and maintained under recommended conditions.

Authentication | 293T cells used for retrovirus production were obtained from ATCC (CRL-3216) and authenticated as described in Roy et al., 2020 (Cell Metabolism, 31(2):250–266.e9). EO-771 breast cancer cells were obtained from ATCC (CRL-3461). B16-F10 melanoma cells expressing OVA (B16-OVA) and MC38-OVA-tdTomato cells were provided and authenticated as reported in Luda et al. (reference to included in bibliography).

Mycoplasma contamination | Cells have been verified as mycoplasma-free using the MycoAlert Mycoplasma Detection Kit (Lonza).

Commonly misidentified lines (See ICLAC register) | No commonly misidentified cell lines were used in this study

Animals and other research organisms

Policy information about studies involving animals; ARRIVE guidelines recommended for reporting animal research, and Sex and Gender in Research

Laboratory animals | This study used the following mouse strains: C57BL/6J (RRID: IMSR_JAX:000664); B6.PL-Thy1a/CyJ (Thy1.1; RRID: IMSR_JAX:000406),

Laboratory animals	B6.SJL-Ptprca Pepcb/BoyJ (CD45.1+), Tg(TcraTcrb)1100Mjb (OT-I; RRID: IMSR_JAX:003831), all ordered from The Jackson Laboratory. The Bdh1fl/fl/Oxct1fl/flCd4-Cre line was generated by crossing Bdh1fl/flCd4-Cre mice22 and Oxct1-floxed mice (provided by Peter Crawford69,70). Bdh1fl/flOxct1fl/flCd4-Cre OT-I mice were generated by crossing Bdh1fl/flOxct1fl/flCd4-Cre mice with the Tg(TcraTcrb)1100Mjb mouse line. All mice were bred and housed under specific pathogen-free conditions at VAI, following approved protocols. Genotyping was conducted using DNA extracted from tail or ear biopsies, with primer sets listed in Key Resources Table. The study included both male and female mice aged 8 to 14 weeks.
Wild animals	No wild animals were used in this study.
Reporting on sex	Baseline immunophenotyping of WT vs DR mice revealed no key sex specific differences in examined immune cell types. Tumor studies used a mix of male and female mice for all experiments with the exception to Fig. 7G (only female).
Field-collected samples	No field collected samples were used in this study
Ethics oversight	Mice were maintained under specific pathogen-free conditions at VAI under approved IACUC protocols

Note that full information on the approval of the study protocol must also be provided in the manuscript.

Plants

Seed stocks	No seed stocks were used in this study.
Novel plant genotypes	No novel plant genotypes were used in this study.
Authentication	No authentication procedures were required as no seed stocks or novel plant genotypes were used in this study

Flow Cytometry

Plots

Confirm that:

☒ The axis labels state the marker and fluorochrome used (e.g. CD4-FITC).

☒ The axis scales are clearly visible. Include numbers along axes only for bottom left plot of group (a 'group' is an analysis of identical markers).

☒ All plots are contour plots with outliers or pseudocolor plots.

☒ A numerical value for number of cells or percentage (with statistics) is provided.

Methodology

Sample preparation	Mice were sacrificed and spleens were obtained—or blood was obtained via submental bleed of live mice—and lymphocytes were isolated using red blood cell (RBC) lysis buffer containing 0.15M NH4Cl, 10 mM KHCO3, and 0.1 mM EDTA, followed by neutralization with 3 volumes of TCM. Lymphocyte suspensions were surface stained with a cocktail of fluorescently-labeled antibodies
Instrument	Flow cytometry was performed on Cytoflex (Beckman Coulter) or Aurora Cytek cytometers and cell sorting on Astrios (Beckman Coulter) or BD FACSAria Fusion cell sorters.
Software	Flow cytometry data was collected using SpectroFlo software (version 3.0.3) from Cytek.<br>Flow cytometry analysis was conducted using FlowJo software (version 10.10.0).
Cell population abundance	Relevant cell populations were isolated by fluorescence-activated cell sorting (FACS) to >99% purity based on surface marker expression or reporter fluorescence. For endogenous populations, $CD8a^{+}P14^{+}$ T cells were sorted using fluorescent antibody staining. For virally transduced populations, the top one-third of GFP^{+} or $Ametrine^{+}$ cells were gated and sorted. Post-sort purity was determined by re-analysis of sorted fractions, confirming >99% enrichment of the target population.

Gating strategy

Total splenocytes were first gated based on forward scatter area (FSC-A) versus side scatter area (SSC-A) to select the overall cell population. Singlets were subsequently identified by gating FSC-A versus forward scatter height (FSC-H). Live cells were gated based on SSC-A versus fixable viability dye exclusion. For example, $CD8^+$ T cells were then identified by gating SSC-A versus CD8 expression, with positive gates set based on clear population separation. For antigen-specific T cells, $CD44^+$ versus $tetramer^+$ (e.g., OVA tetramer) cells were gated from the $CD8^+$ population. PD-1^+ cells were gated based on fluorescence-minus-one (FMO) controls and by referencing internal negative populations (e.g., $CD44^-$ naïve T cells) to define positive boundaries. For intracellular cytokine staining, unstimulated controls were used to define gates for IFNγ and TNF production.

☐ Tick this box to confirm that a figure exemplifying the gating strategy is provided in the Supplementary Information.
