# Supplementary figures and images for: Dietary restriction reprograms CD8+ T cell fate to enhance anti-tumour immunity and immunotherapy responses
Source: Nat Metab. 2025 Dec 9;7(12):2489–509. doi: 10.1038/s42255-025-01415-6 (PMC12727518; doi:10.1038/s42255-025-01415-6)

**Figure 6C**

CD8<sup>+</sup> T Cell Immunoblot

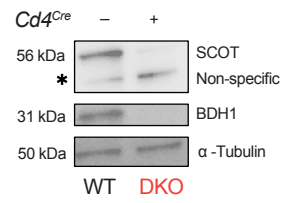

OXCT1 (56kDa)

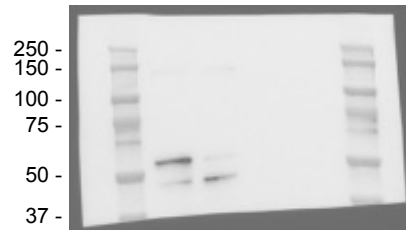

BDH1 (31kDa)

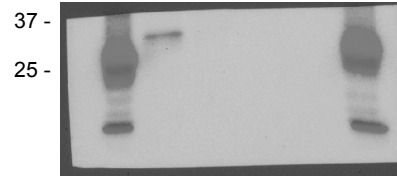

$\alpha$ -Tubulin (50 kDa)

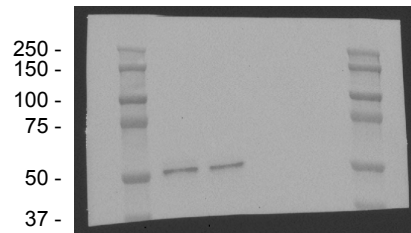

Supplement: Supplementary file 19 — Unprocessed western blots [file 42255_2025_1415_MOESM19_ESM.pdf]
